# Comparative Transcriptomics Analysis and Functional Study Reveal Important Role of High-Temperature Stress Response Gene *GmHSFA2* During Flower Bud Development of CMS-Based F_1_ in Soybean

**DOI:** 10.3389/fpls.2020.600217

**Published:** 2020-12-15

**Authors:** Xianlong Ding, Qingling Guo, Qiang Li, Junyi Gai, Shouping Yang

**Affiliations:** Soybean Research Institute, National Center for Soybean Improvement, Key Laboratory of Biology and Genetic Improvement of Soybean (General, Ministry of Agriculture), State Key Laboratory of Crop Genetics and Germplasm Enhancement, Jiangsu Collaborative Innovation Center for Modern Crop Production, College of Agriculture, Nanjing Agricultural University, Nanjing, China

**Keywords:** soybean, CMS-based F_1_, HT stress, RNA-seq, *HSFA2*

## Abstract

High-temperature (HT) is one of the most important environmental factors that negatively impact the yield of some soybean cytoplasmic male sterility (CMS)-based hybrid (F_1_) combinations. The response of soybean to HT, especially at the male organ development stage, is poorly understood. To investigate the molecular mechanisms of the response from soybean CMS-based F_1_ male organ to HT, a detailed transcriptomics analysis was performed during flower bud development of soybean HT-tolerant and HT-sensitive CMS-based F_1_ combinations (NF_1_ and YF_1_) under normal-temperature and HT conditions. Obvious HT damage was observed by subjecting YF_1_ with HT, such as indehiscent anthers and decreased pollen fertility, whereas the male fertility of NF_1_ was normal. In total, 8,784 differentially expressed genes (DEGs) were found to respond to HT stress, which were mainly associated with anther/pollen wall development, carbohydrate metabolism and sugar transport, and auxin signaling. The quantitative real-time PCR (qRT-PCR) analysis and substance content detection also revealed that HT caused male fertility defects in YF_1_ by altering pectin metabolism, auxin, and sugar signaling pathways. Most importantly, the sugar signaling-*PIF*-auxin signaling pathway may underlie the instability of male fertility in YF_1_ under HT. Furthermore, HT induced the expression of heat shock factor (*HSF*) and heat shock protein (*HSP*) gene families. Overexpression of *GmHSFA2* in *Arabidopsis* can promote the expression of HT protective genes (such as *HSP20*) by binding to the HSE motifs in their promoters, so as to improve the HT tolerance during flowering. Our results indicated that *GmHSFA2* acted as a positive regulator, conferring HT tolerance improvement in soybean CMS-based F_1_. *GmHSFA2* may be directly involved in the activation of male fertility protection mechanism in the soybean CMS-based F_1_ under HT stress.

## Introduction

Temperature is an important ecological factor affecting physiological and biochemical processes in plants. The physiological damage caused by a high-temperature (HT) environment to plants is called HT stress (Puteh et al., 2013). For plants, even an increase of 1°C in the threshold level is considered as HT stress (Teixeira et al., 2013). The development of male organs in plants is extremely sensitive to temperature, and continuous HT stress will cause microspore abortion, anther indehiscence, filament shortening, and abnormal pollen viability or germination (Djanaguiraman et al., 2018; Begcy et al., 2019). In recent years, with the increase of global temperature, HT stress has become a serious factor affecting crop growth and development (Min et al., 2013; Li et al., 2018).

The “cytoplasmic male sterility (CMS)-based” breeding system is composed of the CMS line and its corresponding maintainer line and restorer line, which is one of the most widely used breeding systems in crop hybrid (F_1_) seed production (Chen and Liu, 2014). At present, the “CMS-based” matching system has been successfully applied in rice, maize, soybean, and other crops (Chen and Liu, 2014). In the process of hybridization, the sterility of the male sterile line can be restored by the fertility restorer gene of the male restorer line. Due to the genetic effects of cytoplasmic and nuclear interactions between the CMS line and its restorer line, CMS-based F_1_ is generally more sensitive to the external environment than conventional materials, especially for gametophyte sterile material, in which only about 50% of its CMS-based F_1_ pollen is fertile (Xie, 2008). Under the influence of certain conditions (including HT stress), the percentage of fertile pollen may be greatly reduced for CMS-based F_1_ and eventually fail to develop normal seeds (Xie, 2008).

It has been reported that HT is one of the main factors affecting the difference of CMS-based F_1_ fertility restoration in some plants, such as cotton and rice (Zhao et al., 2009; Zhang et al., 2019). Continuous HT stress resulted in insufficient anther dehiscence, decreased pollen survival rate, and finally decreased yield of CMS-based F_1_. In general, soybeans begin to bloom in late July. However, extreme HT frequently occurred in July and August in the Huanghuai region and South China, the main producing areas of summer-sown soybean in China. Similar to rice and cotton, the male fertility of soybean CMS-based F_1_ is also unstable under HT (Nie et al., 2017).

In recent years, it has been reported that fertility-enhancing genes and DNA methylation are involved in the fertility regulation of cotton CMS-based F_1_ (Wang, 2019; Zhang et al., 2019). Wang (2019) found that pollen fertility of cotton CMS-based F_1_ is related to the restorer gene and fertility-enhancing gene such as *GST* (Zhu, 2005). Under the same nuclear genetic background of the restorer gene, the restorer line with strong adaptability can be selected using different ecological environments (Wang, 2019). Zhang et al. (2019) found that HT-induced DNA methylation maintained the dynamic balance of ATP synthesis and ROS production by upregulating the expression of mitochondrial respiratory chain-related genes, so as to ensure the normal fertility recovery ability of the cotton CMS-D2 system under HT stress. However, no study has focused on the molecular mechanism of HT-induced male fertility instability in soybean CMS-based F_1_. In order to better understand the molecular mechanism of difference in male fertility restoration of soybean CMS-based F_1_ under HT stress, anther/pollen morphology observation, RNA sequencing (RNA-seq), physiological and biochemical determination, and gene functional verification were performed. Cytological observation showed that soybean HT-sensitive CMS-based F_1_ was mainly characterized by anther indehiscence and decreased pollen fertility under HT stress. Based on the analysis of differentially expressed genes (DEGs) and differential metabolites, we found that genes or substances related to anther/pollen wall development and auxin metabolism, carbohydrate metabolism, sugar transport, transcription factors (TFs), and heat shock proteins (*HSP*) may be involved in the fertility regulation of soybean CMS-based F_1_ under HT. Most importantly, it was found that *GmHSFA2* can regulate *HSP* and *galactinol synthase* (*GolS*)-related genes to improve HT tolerance of plants.

## Materials and Methods

### Plant Materials and HT Treatment

Two soybean CMS-based F_1_ combinations of the CMS system with different degrees of HT stress tolerance in the field were used in this study, namely, NF_1_ and YF_1_, which are tolerant and sensitive to HT stress, respectively. The hybridization of the CMS line NJCMS1A and its restorer lines N4608 and YY6 was carried out in the field at Dangtu Experimental Station (National Center for Soybean Improvement, Nanjing Agricultural University, Dangtu, Anhui, China) in the summer of 2017. And the F_1_ seeds of (NJCMS1A × N4608) and (NJCMS1A × YY6) were harvested in the autumn, which were designated as NF_1_ and YF_1_, respectively, in this study. The plants were grown in illuminated incubators (RXZ-430D, Ningbo Jiangnan, Ningbo, China) at 26 ± 1/20 ± 1°C (day/night) with a 12-h light/12-h dark photoperiod during seedling. The flowering plants were grown in an illuminated incubator at 30°C/24°C (day/night) considered as a normal-temperature (NT) condition. For temperature gradient treatment, three individual flowering plants (R1 stage) of each combination were incubated at 38/32°C and 34/28°C (day/night) for 7 days. During HT treatment, the flowering plants (R1 stage) were incubated at 38/32°C in an illuminated incubator. Because it is very difficult to judge the precise development stage of pollen from the appearance of the flower buds in soybean as described previously (Ding et al., 2016), after HT treatment for 7 days, flower buds of different sizes were collected from NF_1_ and YF_1_ plants under NT and HT, respectively, and then immediately frozen in liquid nitrogen and stored at −80°C for RNA isolation. To analyze the expression patterns of *GmHSFA2* (*Glyma.14G096800*) and *GmHSP20a* (*Glyma.12G013100*) genes, flowering plants from N4608 were initially exposed to 40°C for 7 days of HT treatment and then transferred to NT (30°C) for recovery. Flower buds of different sizes were sampled at time points of 0, 1st, 3rd, 5th, 7th day and 1 day after recovery. Flower buds of each genotype were collected from three individual plants as three independent biological replicates for NF_1_NT, YF_1_NT, NF_1_HT, YF_1_HT, and N4608.

The *Arabidopsis thaliana* Columbia (Col-0) ecotype was used as wild-type (WT) control. The *35S:GmHSFA2*, *pGmHSFA2:GUS*, and *35S:GmHSP20a* transgenic plants were all in the Col-0 background. The seeds were vernalized for 2 days at 4°C and then cultivated on a prefertilized soil mixture (nutritional soil, perlite, and vermiculite at a 3:1:1 ratio) at 23°C with long-day conditions (16 h light/8 h dark) in an illuminated incubator (RXZ-430D, Ningbo Jiangnan, Ningbo, China). To evaluate the HT damage on inflorescence and the expression levels of *GmHSFA2* downstream regulatory genes under HT stress, three *35S:GmHSFA2* transgenic lines and WT were exposed to HT stress at 45/40°C (day/night) for 3 days. The HT treatment on male fertility was performed as Kim et al. (2001) described. The *35S:GmHSFA2* and *35S:GmHSP20a* transgenic plants (two lines for each transgenic type) and WT were held in an illuminated incubator (RXZ-430D, Ningbo Jiangnan, Ningbo, China) at 42°C for 4 h and then transferred to normal growth conditions. All types were grown at 23°C as control.

### RNA Isolation and cDNA Library Construction

Total RNA from the flower buds of NF_1_NT, YF_1_NT, NF_1_HT, and YF_1_HT (three independent biological replicates for each genotype) was extracted using the TRIzol reagent (Invitrogen, Carlsbad, CA, United States) according to the manufacturer’s protocol. In order to obtain mitochondrial and chloroplast-related genes, this study refers to the cDNA library construction of prokaryote, considering that the plant mitochondrial and chloroplast genomes are similar to its ring genome. So after total RNA was extracted, sample mRNA was enriched by removing rRNA by a Ribo-Zero^TM^ Magnetic Kit (Epicentre). Then the enriched mRNA was fragmented into short fragments using a fragmentation buffer and reverse transcribed into cDNA with random primers. A second-strand cDNA was synthesized by DNA polymerase I, RNase H, dNTP, and buffer. Then the cDNA fragments were purified with a QIAquick PCR extraction kit, end repaired, poly(A) added, and ligated to Illumina sequencing adapters. The ligation products were size selected by agarose gel electrophoresis, PCR amplified, and sequenced using Illumina HiSeq^TM^ 2500 by Gene *Denovo* Biotechnology Co. (Guangzhou, China).

### Data Analysis of RNA-Seq

Raw reads were filtered to obtain high-quality reads by removing reads containing adapters or more than 10% of unknown nucleotides (N) and more than 50% of low-quality (*Q*-value ≤ 20) bases. The rRNA mapped reads were removed by a short-reads alignment tool Bowtie 2 (Langmead and Salzberg, 2012). Clean reads (the rRNA removed reads) were subsequently aligned with the soybean Williams 82 reference genome (Wm82.a2.v1) using TopHat2 (version 2.0.3.12, Kim et al., 2013). Gene abundances were quantified by software RSEM (Li and Dewey, 2011), and the gene expression level was normalized by using the fragments per kilobase of transcript per million mapped reads (FPKM) method (Mortazavi et al., 2008).

Subsequent data were analyzed using repeated correlation analysis (RCA) and principal component analysis (PCA). The correlation coefficient between the two replicas was calculated to evaluate repeatability between samples. The closer the correlation coefficient gets to 1, the better the repeatability between two parallel experiments. The PCA was performed with R package models^1^; it is largely used to reveal the relationship of NF_1_NT, YF_1_NT, NF_1_HT, and YF_1_HT. To identify DEGs across samples or groups, the edge R package (see text footnote 1) was used. Only genes with | Log_2_FC (fold change)| ≥ 1 and false discovery rate (FDR) ≤ 0.05 were identified as significant DEGs. Gene Ontology (GO) enrichment analysis provides all GO terms that are significantly enriched in DEGs compared to genomic backgrounds and maps all DEGs to GO terms in the GO database.^2^ GO terms with FDR ≤ 0.05 were considered to be significantly enriched. The Kyoto Encyclopedia of Genes and Genomes (KEGG) pathway enrichment was performed in the KEGG database web server^3^ (Kanehisa et al., 2008). Pathways with FDR ≤ 0.05 were defined as significantly enriched pathways in DEGs.

### Plant Transformation

Full-length CDS clones of the *GmHSFA2* (*Glyma.14G096800*) and *GmHSP20a* (*Glyma.12G013100*) genes were obtained from SoyBase.^4^ Two overexpression constructs were generated by inserting the full-length *GmHSFA2* and *GmHSP20a* CDS fragments into the binary vector *pCAMBIA3301-26* after the CaMV 35S promoter, using a one-step cloning kit (Vazyme, Nanjing, China) and designated as *35S:GmHSFA2* and *35S:GmHSP20a*, respectively. The promoter of *GmHSFA2* (2,000 bp) was amplified by PCR using N4608 DNA and replaced the 35S promoter of *pCAMBIA3301-GUS* using *Hin*dIII and *Nco*I digestion, resulting in a plasmid of *pGmHSFA2:GUS*. All the above overexpression vectors were introduced into *Agrobacterium tumefaciens* strain EHA105 via the freeze–thaw method. *Agrobacterium*-mediated floral dip method was used for *Arabidopsis* transformation (Clough and Bent, 1998). The specific primers used for CDS and promoter cloning are given in Supplementary Table 1. Transgenic plants (T_0_, T_1_, T_2_, and T_3_) were screened by the Murashige and Skoog medium glufosinate (20 mg/L).

### GUS Staining and Plant Trait Investigation

The inflorescence of *pGmHSFA2:GUS* plant materials was GUS stained following the protocol of Jefferson et al. (1987). The morphology of anthers from opened flowers of soybean and *Arabidopsis* was observed under an Olympus CX31 microscope (Japan). Pollen viability of soybean and *Arabidopsis* was analyzed by I_2_-KI staining (Nie et al., 2019) and Alexander’s staining (Ding et al., 2020), respectively. The stamen and pistil length of *Arabidopsis* was measured with the cellSens software (Olympus, Japan). Nine flower buds/flowers of each genotype/line were collected from three individual plants to measure the length of stamen/pistil and observe the fertility of pollen. Student’s *t*-test was performed to compare the trait differences between the experimental group and the control group.

### Quantitative Real-Time PCR Analysis

The quantitative real-time PCR (qRT-PCR) was used to validate the gene expression levels in soybean and *Arabidopsis*. All primers (Supplementary Table 1) were designed based on the mRNA sequences and synthesized commercially (General Biosystems, Chuzhou, China). Total RNA from the same soybean samples that constructed the cDNA library was used for the validation of RNA-seq. According to the procedures provided in the HiScript Q RT SuperMix for the qPCR kit (**+**gDNA wiper, Vazyme, Nanjing, China), 1 μg of total RNA was reverse-transcribed using an Oligo(dT) primer. The mRNA qRT-PCR analysis was carried out using AceQ qPCR SYBR Green Master Mix (Vazyme, Nanjing, China) on a Bio-Rad CFX96 instrument (CFX96 Touch, Bio-Rad, United States). For *Arabidopsis*, all reactions were run with three independent biological replicates, each comprising three individual plants, and *AtActin* (accession number: *NM_001338359.1*) was used as internal control genes. For soybean, *GmTubulin* (accession number: *NM_001252709.2*) was used as internal control genes. The NF_1_ and WT under the NT condition were used as the control in qRT-PCR experiments on soybean and *Arabidopsis*, respectively. The relative expression levels of the genes were quantified using the 2^–ΔΔCt^ method (Livak and Schmittgen, 2001). Student’s *t*-test was performed to compare mRNA expression differences between the experimental group and the control group.

### Substance Content and Enzyme Activity Assays

Flower buds of different sizes were collected from NF_1_ and YF_1_ plants (three independent biological replicates for each genotype) under NT and HT for substance content and enzyme activity assays. The contents of Suc, Glc, starch, and IAA were determined on a UV–vis spectrophotometer (EU-2600D, Onlab, Shanghai, China) using a Suc assay kit (Jiancheng, Nanjing, China), Glc assay kit (Sinobestbio, Shanghai, China), starch assay kit (Sinobestbio, Shanghai, China), and IAA assay kit (Mallbio, Nanjing, China), respectively, by following the manufacturer’s protocol. The pectinase activity was measured at 540 nm on a microplate reader (SpectraMax iD5, Molecular Devices, United States) using the pectinase assay kit (Sinobestbio, Shanghai, China) by following the manufacturer’s protocol. Three independent biological replicates were assayed, and one-way ANOVA and Duncan’s test were performed for statistical analysis.

### Subcellular Localization and Yeast One-Hybrid Assay

The open reading frame (ORF) (after removal of the stop codon) of *GmHSFA2* was integrated into the 5′ end of the green fluorescent protein (*GFP*) coding region in the *pCAMBIA3301-GFP* vector using *Bgl*II digestion, resulting in a plasmid of *GmHSFA2-GFP*. Both *GmHSFA2-GFP* and *pCAMBIA3301-GFP* (control) constructs were transformed into tobacco (*Nicotiana benthamiana*) leaves according to the protocol of Sparkes et al. (2006). The treated seedlings of tobacco were grown at 23°C with long-day conditions (16 h light/8 h dark) in an illuminated incubator (RXZ-430D, Ningbo Jiangnan, Ningbo, China) for 3 days and then observed under a confocal laser scanning microsystem LSM780 (Carl Zeiss, Jena, Germany) with 488-nm excitation wavelengths.

The direct interaction between *GmHSFA2* and the promoter of *GmHSP20a* was detected by the yeast one-hybrid (Y1H) assay system. Four tandem *cis*-acting HSE motifs present in the promoter region of *GmHSP20a* were amplified by PCR using N4608 DNA and integrated into the *PAbAi* vector, yielding *pAbAi-pGmHSP20a* as bait, while the full-length CDS of *GmHSFA2* was amplified from the *35S:GmHSFA2* vector and inserted into a *pGADT7* vector, yielding a *pGADT7-GmHSFA2* construct as prey. The primers used are listed in Supplementary Table 1. The *pGBKT7-pGmHSP20a* was first introduced into the Y1H gold yeast (Clontech) and cultured on SD/–Ura and SD/–Ura/A medium for self-activating detection. After that, the *PGADT7*, negative control, and positive control vectors were introduced and cultured on SD/–Ura/A for spot assay.

## Results

### Characterization of Soybean CMS-Based F_1_ Male Fertility Under HT Stress

**FIGURE 1:**
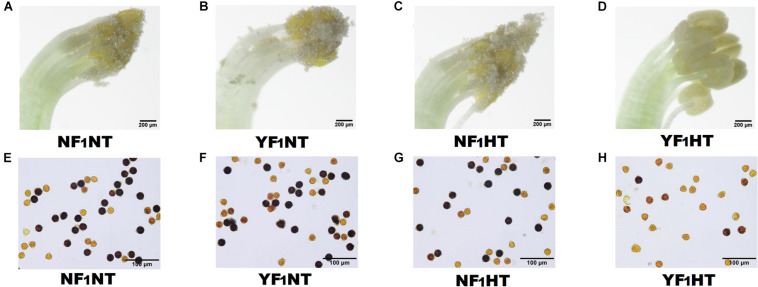
Phenotypic characteristics of soybean male fertility under NT and HT conditions. **(A–D)** Soybean anthers from NF_1_ and YF_1_ under NT and HT conditions. **(E–H)** Soybean pollens from NF_1_ and YF_1_ under NT and HT conditions. The sterile pollen was stained bright yellow by I_2_-KI solution.

To explore the mechanism of male fertility instability under HT stress, two soybean CMS-based F_1_ combinations were used in this study, namely, NF_1_ (HT tolerant) and YF_1_ (HT sensitive) (Supplementary Figure 1). There was no difference in male fertility between the two combinations under NT (30°C) according to gradient temperature treatment (Supplementary Figures 2A,B and Figure 1). However, obvious HT damage was observed by subjecting YF_1_ to gradient temperatures (30, 34, and 38°C, such as worse anther dehiscence and gradually decreasing pollen fertility (Supplementary Figures 2A,B). When the temperature reached 38°C (HT treatment in this study), the male fertility of YF_1_ was significantly affected, and YF_1_ finally displayed forms of anther indehiscence and decreased pollen fertility, while NF_1_ performed normally (Figure 1).

### Transcriptomics Analysis in Flower Buds of NF_1_ and YF_1_ Under HT Stress

To gain insight into the molecular mechanism of male fertility reduction in soybean HT-sensitive CMS-based F_1_ under HT stress, RNA-seq was performed for both HT-tolerant and HT-sensitive F_1_ during flower bud development under NT and HT conditions. A total of 50.41 million raw reads were generated from 12 samples with an average read length of 150 bp (Table 1). After removal of reads containing adapters, poly(N) greater than 10%, and low-quality sequences, an average of 6.11-Gb clean data were obtained for each sample (Table 1). After removal of reads mapped on rRNA, 89.70–90.57% of clean reads were mapped to the soybean reference genome (Gmax_275_Wm82.a2.v1) (Table 1).

**TABLE 1:** Data statistics of cDNA libraries from NF_1_NT, YF_1_NT, NF_1_HT, and YF_1_HT.

Sample	Raw reads	Raw reads (bp)	HQ clean reads	HQ clean data (bp)	Clean bases (Gb)	Q20 (%)	Q30 (%)	GC (%)	Mapping ratio (%)
NF_1_NT-1	38467484	5770122600	37832070	5596841295	5.60	98.32	94.58	43.82	89.70
NF_1_NT-2	59467424	8920113600	58497786	8655405125	8.66	98.44	94.90	43.77	90.43
NF_1_NT-3	40597054	6089558100	39934094	5908373737	5.91	98.39	94.79	43.85	90.24
YF_1_NT-1	42872316	6430847400	42115742	6224542494	6.22	98.36	94.68	44.14	90.57
YF_1_NT-2	30496662	4574499300	29953826	4427793915	4.43	98.28	94.47	43.95	90.49
YF_1_NT-3	40171536	6025730400	39432772	5824635146	5.82	98.41	94.85	44.02	90.61
NF_1_HT-1	38467484	5770122600	37832070	5596841295	5.60	98.32	94.58	43.82	89.70
NF_1_HT-2	59467424	8920113600	58497786	8655405125	8.66	98.44	94.90	43.77	90.43
NF_1_HT-3	40597054	6089558100	39934094	5908373737	5.91	98.39	94.79	43.85	90.24
YF_1_HT-1	42872316	6430847400	42115742	6224542494	6.22	98.36	94.68	44.14	90.57
YF_1_HT-2	30496662	4574499300	29953826	4427793915	4.43	98.28	94.47	43.95	90.49
YF_1_HT-3	40171536	6025730400	39432772	5824635146	5.82	98.41	94.85	44.02	90.61
Average	42012079.33	6301811900	41294381.67	6106265285	6.11	98.37	94.71	43.93	90.34

*HQ, high quality. Sequence length was 2 × 150 bp, length of each read was 150 bp using double end sequencing.*

Principal component analysis was used to analyze the relationship between two genotypes under NT and HT conditions. The first principal component (PC1) accounted for 78.2% of the variance, and the second principal component (PC2) accounted for 12.0% of the variance (Supplementary Figure 3). With the exception of NF_1_HT, the three biological replicas in each group were clustered closely together. In general, NF_1_ and YF_1_ were significantly different under NT and HT conditions. As shown in Supplementary Figure 4, the correlation coefficients (*R*^2^) between the biological replicates of each group were greater than 0.96 and close to 1, indicating that each group had good repeatability.

### Identification of DEGs in Response to Heat Stress

**FIGURE 2:**
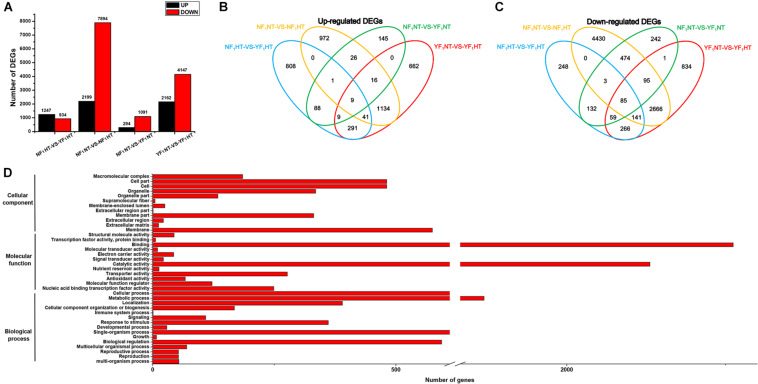
Analysis of DEGs between NF_1_ and YF_1_ under NT and HT conditions. **(A)** Number of DEGs that were up- and down-regulated under NT and HT conditions. **(B,C)** Venn diagram of common up- and down-regulated DEGs under NT and HT conditions. **(D)** GO classification of DEGs. The *x*-axis indicates the number of DEGs, and the *y*-axis indicates the GO groups.

Significantly DEGs were screened between the different samples with the criteria of fold change ≥ 2 and FDR ≤ 0.05. To determine the genes that were differentially expressed between two genotypes under NT and HT conditions, four comparisons (NF_1_NT vs YF_1_NT, NF_1_NT vs NF_1_HT, YF_1_NT vs YF_1_HT, and NF_1_HT vs YF_1_HT) were performed. Under the NT condition, a total of 1,385 (294 upregulated and 1,091 downregulated) DEGs were identified for the comparison of NF_1_NT vs YF_1_NT (Figure 2A). After HT stress, 13,491 genes were differentially expressed in different comparisons. Among these, a total of 10,093 (2,199 upregulated and 7,894 downregulated) and 6,309 (2,162 upregulated and 4,147 downregulated) DEGs were identified for the comparisons of NF_1_NT vs NF_1_HT and YF_1_NT vs YF_1_HT, respectively (Figure 2A). We identified 4,187 (1,200 upregulated and 2,987 downregulated) DEGs that were in common among these two pairs. A total of 2,181 (1,247 upregulated and 934 downregulated) DEGs were identified for the pair of NF_1_HT vs YF_1_HT. The Venn diagram showed that the groups NF_1_NT vs YF_1_NT and NF_1_HT vs YF_1_HT had only 386 (107 upregulated and 279 downregulated) DEGs in common. However, the DEGs under HT stress accounted for 56.45% of the total DEGs (3,180 DEGs) of these two combinations. This indicated that most DEGs had differential expression changes in response to HT stress. Based on the Venn diagram, we found that 4,519 DEGs showed the same expression pattern between NF_1_ and YF_1_ and that the remaining 9,359 DEGs were upregulated or downregulated in different comparisons under HT stress (Figures 2B,C). Twelve DEGs were randomly selected for qRT-PCR verification, and the coincidence rate between qRT-PCR results and RNA-seq data was 100% (Supplementary Figure 5), supporting the reliability of expression patterns revealed by RNA-seq.

### Functional Classification of DEGs in Response to Heat Stress

Among the 9,359 DEGs between the NT and HT samples, 2,244 upregulated and 5,965 downregulated genes were identified, and the other 575 DEGs were upregulated or downregulated in different combinations at the same time. In order to understand the potential functions in the list of DEGs, all 8,784 DEGs were further analyzed for GO functional annotations. The results revealed that 4,482 DEGs could be classified into 39 GO terms: 3,024 DEGs participated in biological processes, 4,034 DEGs had molecular functions, and 1,018 DEGs had cellular components (Figure 2D and Supplementary Tables 2–4). At the biological process level, the DEGs are enriched into 23 biological processes (*p*.adjust ≤ 0.05), including pollination (GO:0009856), reproduction (GO:0000003), phosphorylation (GO:0016310), response to oxidative stress (GO:0006979), and oxidation-reduction process (GO:0055114). In particular, we also observed two DEGs in the GO terms response to heat (GO:0009408) and response to temperature stimulus (GO:0009266). Similarly, large numbers of DEGs were also enriched in the molecular function and cellular component, including pectinesterase activity (GO:0030599), peroxidase activity (GO:0004601), antioxidant activity (GO:0016209), cell wall (GO:0005618), and membrane (GO:0016020).

**FIGURE 3:**
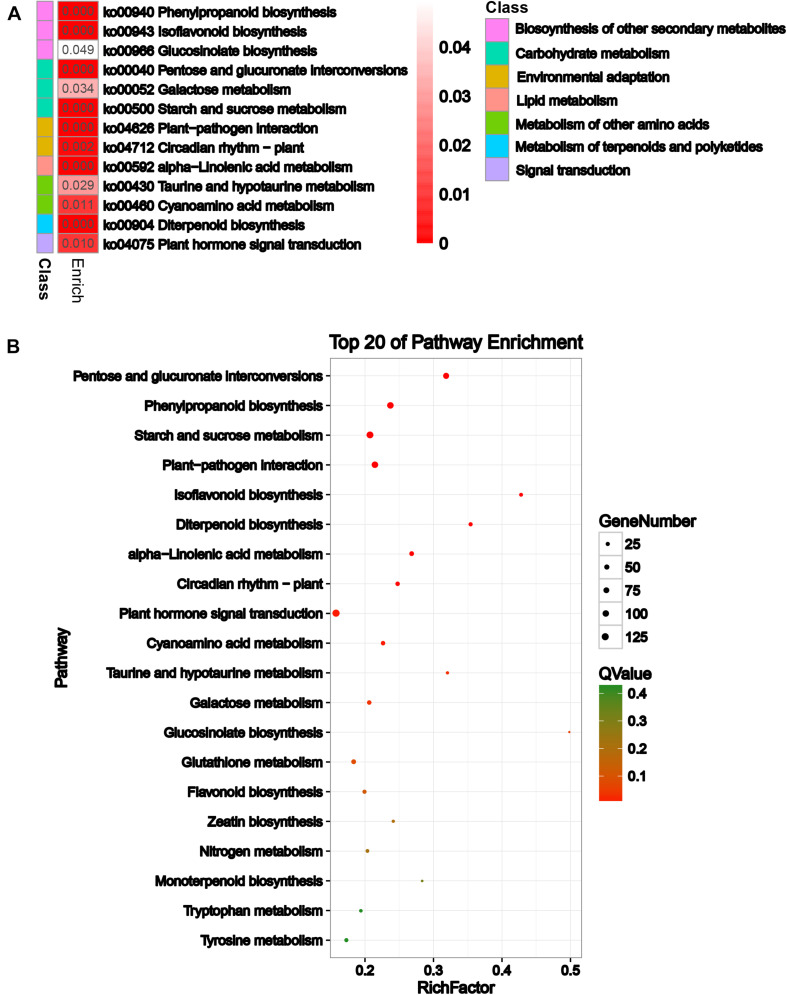
KEGG enrichment analysis of the DEGs. **(A)** KEGG analysis with heat map. **(B)** Top 20 of pathway enrichment. The *x*-axis indicates the rich factor corresponding to each pathway, and the *y*-axis indicates name of the KEGG pathway. The color of the point represents the *P*-values of the enrichment analysis. The size and color of bubbles represent the number and degree of enrichment of DEGs, respectively.

To explore the biological pathways on the reproductive development of soybean CMS-based F_1_ on which HT has an important influence, KEGG pathway analysis was further performed for these DEGs. A total of 13 significant KEGG pathways (*Q*-value ≤ 0.05) were enriched for 1,409 DEGs (Supplementary Table 5 and Figure 3A), including pentose and glucuronate interconversions, phenylpropanoid biosynthesis, and starch and sucrose metabolism (Figure 3B). Most importantly, HT stress-induced DEGs were mostly enriched in pentose and glucuronate interconversions, starch and sucrose metabolism, phenylpropanoid biosynthesis, flavonoid biosynthesis, and circadian rhythm–plant pathways, which belong to carbohydrate metabolism, biosynthesis of other secondary metabolites, and environmental adaptation classes, respectively, for the comparisons NF_1_HT vs YF_1_HT and YF_1_NT vs YF_1_HT (Supplementary Figures 6A,D). This is consistent with the male sterile phenotype of YF_1_ under HT.

### HT Caused Anther Defects by Altering Anther/Pollen Wall Development

**FIGURE 4:**
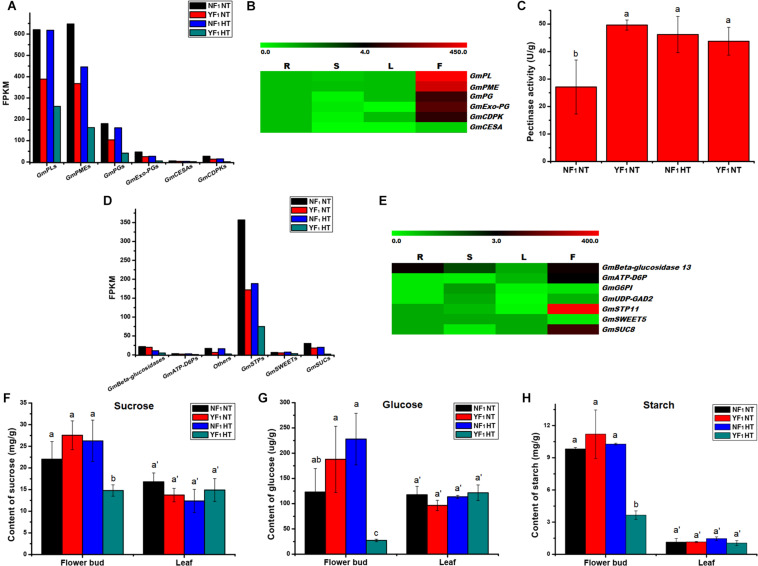
Effect of HT on anther/pollen wall development, carbohydrate metabolism and sugar transport. **(A)** DEGs related to anther/pollen wall development in RNA-seq data. **(B)** Heat map of the DEGs related to anther/pollen wall development in four different tissues. The color scale represents the relative transcript abundance of the DEGs in four soybean tissues. The heat map was conducted using MeV 4.9 software. The FPKM values were obtained from the RNA-seq data in Phytozome v12.0. R, root; S, stem; L, leaf; F, flower. **(C)** Pectinase activity in flower buds of NF_1_ and YF_1_ under NT and HT conditions (*n* = 3). Data are presented as means ± standard deviation (SD) from independent biological replicates. Values with different letter indicates statistical differences (one-way ANOVA, Duncan, *P* < 0.05). **(D)** DEGs related to carbohydrate metabolism and sugar transport in RNA-seq data. **(E)** Heat map of the DEGs related to carbohydrate metabolism and sugar transport in four different tissues. The color scale represents the relative transcript abundance of the DEGs in four soybean tissues. The heat map was conducted using MeV 4.9 software. The FPKM values were obtained from the RNA-seq data in Phytozome v12.0. R, root; S, stem; L, leaf; F, flower. **(F–H)** Suc, glc, and starch contents in flower buds and leaves of NF_1_ and YF_1_ under NT and HT conditions (*n* = 3). Data are presented as means ± standard deviation (SD) from independent biological replicates. Values with different letter indicates statistical differences (one-way ANOVA, Duncan, *P* < 0.05).

Based on the expression level, some enzyme genes involved in pectin metabolism were identified, which included *pectate lyase* (*PL*), *pectin methylesterase* (*PME*, also named *pectinesterase*), *polygalacturonase* (*PG*), and *exopolygalacturonase* (*Exo-PG*). Eleven *PL*s, 21 *PME*s, 17 *PG*s, and 4 *Exo-PG*s were downregulated in flower buds of YF_1_ compared with those of NF_1_ under the HT condition (Figure 4A and Supplementary Table 6). The results also showed lower expressions of *PL*s, *PME*s, *PG*s, and *Exo-PG*s in flower buds of YF_1_ compared with those of NF_1_ under the NT condition (Figure 4A and Supplementary Figures 7A–D). Most importantly, RNA-seq data in Phytozome v12.0 showed that these four type genes were highly expressed in flowers of soybean (Figure 4B). This indicated that the pectinase activity of YF_1_ anthers was defective under the HT condition, which led to abnormal formation of the anther cell wall and finally affected anther dehiscence (Figure 1D). To further confirm this result, the pectinase activity was assessed under NT and HT conditions (Figure 4C). However, pectinase activity in the YF_1_ decreased only slightly compared with that in NF_1_ under HT, which may be due to the pectinase being composed of *PG*, *PL*, and *PME* (Li et al., 2019) that have different activities in the pollen-related tissues and need to be further studied.

The RNA data and qRT-PCR also revealed that three *cellulose synthase proteins* (*GmCESA*s) were downregulated in flower buds of YF_1_ compared with those of NF_1_ under the HT condition (Supplementary Figure 7E), and they were also involved in the pollen wall development of plants (Wang et al., 2011). In addition, three *pollen-specific protein* (*GmCDPK*) DEGs were downregulated in flower buds of YF_1_ under both NT and HT conditions (Supplementary Figure 7F). These results indicated that pectinase, cellulose, and *CDPK* are associated with anther defects in YF_1_ under HT.

### Carbohydrate Metabolism and Sugar Transport in Flower Buds of Soybean HT-Sensitive CMS-Based F_1_ Were Disrupted Under HT

A lot of DEGs involved in carbohydrate metabolism during soybean CMS-based F_1_ flower bud development under HT were found. Among them, there were 79, 117, and 31 DEGs that participated in pentose and glucuronate interconversions, starch and sucrose metabolism, and galactose metabolism pathways, respectively (Supplementary Table 5 and Figure 3). Further analysis indicated that many genes related to carbohydrate metabolism and sugar transport were downregulated, such as *PL*s, *PME*s, *PG*s, *Exo-PG*s, *beta-glucosidase 13*, *ATP-dependent 6-phosphofructokinase 7* (*ATP-D6P7*), *UDP-glucuronic acid decarboxylase 2* (*UDP-GAD2*), *sugar transport protein 11* (*STP11*), *bidirectional sugar transporter SWEET5* (*SWEET5*), and *sucrose transport protein SUC8-like* (*SUC8*) (Supplementary Tables 6, 7). This result was also confirmed by qRT-PCR analysis (Supplementary Figures 7, 8, and Figures 4A,D). Most importantly, most of them were highly expressed in flowers of soybean (Figures 4B,E).

Carbohydrate analysis revealed that sucrose (Suc) and glucose (Glc) accumulation in flower buds of YF_1_ was reduced compared with NF_1_ under HT (Figures 4F,G). Interestingly, Suc and Glc content in either NF_1_ or YF_1_ leaves showed no difference under HT compared with NT (Figures 4F,G). Based on starch content detection, starch accumulation in YF_1_ flower buds was also lower than that in NF_1_ flower buds under HT (Figure 4H). All these results revealed that abnormal carbohydrate transport and accumulation affected pollen development, which was consistent with the results of KEGG analysis and reduction of pollen fertility in YF_1_HT.

### HT Caused Instability of Male Fertility in YF_1_ by Altering Auxin Signaling

**FIGURE 5:**
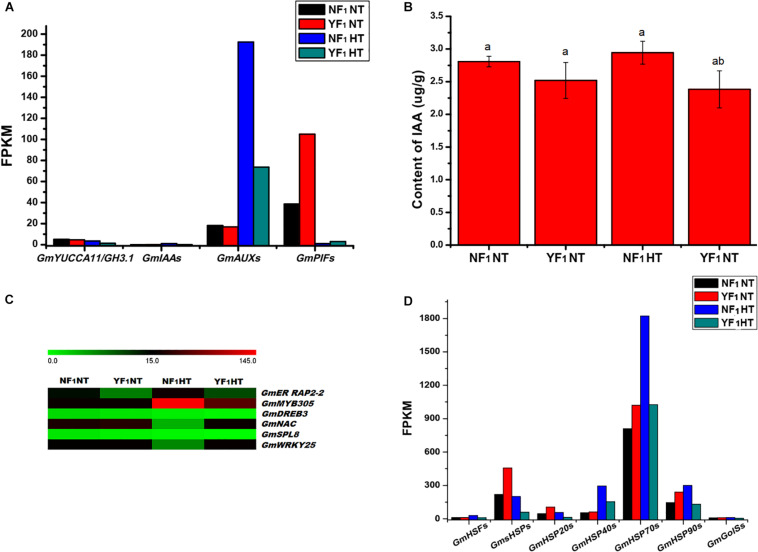
Effect of HT on auxin signaling, transcription factors, *HSP* and *GolS*. **(A)** DEGs related to auxin signaling in RNA-seq data. **(B)** IAA content in flower buds of NF_1_ and YF_1_ under NT and HT conditions (*n* = 3). Data are presented as means ± SD from independent biological replicates. Values with different letter indicates statistical differences (one-way ANOVA, Duncan, *P* < 0.05). **(C)** Heat map of the DEGs related to transcription factors in flower buds of NF_1_ and YF_1_ under NT and HT conditions. The color scale represents the relative transcript abundance of the DEGs in flower bud of soybean. The heat map was conducted using MeV 4.9 software. **(D)** DEGs related to *HSF*, *HSP*, and *GolS* in RNA-seq data.

During soybean CMS-based F_1_ flower bud development under HT, many DEGs were found to be involved in plant hormone signal transduction (Supplementary Table 5). Among them, genes encoding proteins involved in auxin biosynthesis (*YUCCA11* and *GH3.1*), auxin response protein (*IAA29*), and auxin-induced genes (except for *AUX10A5* and *AUXX15*) were downregulated (Supplementary Figure 9). In addition, the expression of IAA regulator *PHYTOCHROME-INTERACTING FACTOR* genes (*PIF1* and *PIF4*) were upregulated (Supplementary Table 8 and Figure 5A). Furthermore, the concentration of endogenous IAA in YF_1_ flower buds was lower than that of NF_1_ under HT (Figure 5B). These results suggested that the reduction in auxin concentration is caused by a decrease in auxin metabolism gene expression, which may lead to anther defects such as anther indehiscence in YF_1_ under HT afterward.

### TFs and *HSP* May Participate in the Male Fertility Regulation of Soybean CMS-Based F_1_ Under HT

Our transcriptomics analysis indicated that numerous HT-responding genes encoding TF are involved in heat signal transduction, such as *heat shock factor* (*HSF*), *ethylene-responsive TF RAP2-2* (*ER RAP2-2*), *myb-related protein 305* (*MYB305*), *dehydration-responsive element-binding protein 3* (*DREB3*), *NAC*, *squamosa promoter-binding-like protein 8* (*SPL8*), and *WRKY25* (Figure 5C). As shown in Supplementary Figure 10, both transcriptomics and qRT-PCR analyses indicated that *ER RAP2-2*, *MYB305*, *DREB3*, and *SPL8* were downregulated in YF_1_HT compared with NF_1_HT, and *NAC* and *WRKY25* were activated by HT in YF_1_.

Remarkably, the rapid response to heat triggered downregulation of a substantial number of *HSF* and *HSP* genes in YF_1_HT (Figure 5D). The results showed that 55 DEGs about *HSF*s and *HSP*s were identified in the NT and HT comparison (Supplementary Table 9 and Supplementary Figure 11). Five *GmHSF*s, namely, four *HSFA* and one *HSFB* genes, were induced in NF_1_ but repressed in YF_1_ under HT. In this study, a total of 50 *GmHSP* genes were identified to be upregulated in NF_1_HT, including 25 small *GmHSP* (*GmsHSP*), 6 *GmHSP20*, 8 *GmHSP40* (DnaJ protein, Georgopoulos et al., 1980), 8 *GmHSP70*, and 3 *GmHSP90* genes.

### Both *GmHSFA2* and Its Downregulated Gene *GmHSP20a* Overexpression Conferred Tolerance to HT Stress During Flowering in *Arabidopsis*

**FIGURE 6:**
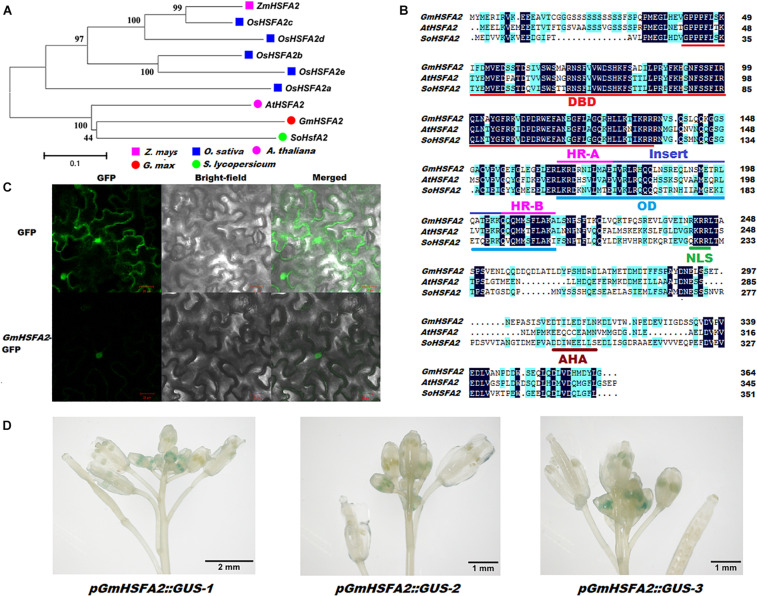
Identification of *GmHSFA2*. **(A)** Phylogenetic tree analysis of *HSFA2* in five plant species. The phylogenetic tree was constructed using MEGA 5.02 based on the Neighbor-joining (NJ) method. Bootstrap values in percentage (1000 replicates) are labeled on the nodes. **(B)** Amino acids sequence comparison and conserved domains of *GmHSFA2* and its closest orthologs *AtHSFA2* and *SoHSFA2* in *Arabidopsis* and tomato, respectively. Sequence alignment was performed with DNAMAN. Dark blue and light blue regions indicate identical and similar amino acids among the three sequences, respectively. DBD, DNA-binding domain; OD, oligomerization domain; HR, hydrophobic heptad repeats; NLS, nuclear localization signal; AHA motif, activator motif. **(C)** Subcellular localization of *GmHSFA2*. **(D)** *pGmHSFA2:GUS* expression pattern in inforescence with developing flower buds during flowering in *Arabidopsis*.

According to the RNA-seq and qRT-PCR analyses, *GmHSFA2* (*Glyma.14G096800*) was induced and inhibited by HT in NF_1_ and YF_1_, respectively (Supplementary Table 9). Its role in HT response was further analyzed. Bioinformatics analysis showed that *GmHSFA2* had high sequence identity with *AtHSFA2* and *SoHSFA2*, which contained a 1,095-bp ORF and predicted to encode 364 amino acids (Figures 6A,B). The alignment revealed that the *GmHSFA2* has the typical domains of *HSFA2*, including a conserved DNA binding domain (DBD), an oligomerization domain (OD) with two adjacent hydrophobic heptad repeats (HR-A/B), a nuclear localization signal (NLS), and an AHA motif (Figure 6B). Subcellular localization analysis showed that the *35S:GmHSFA2-GFP* fusion protein was exclusively localized in the nucleus, which was consistent with the predicted NLS domain between the OD and AHA motif (Figures 6B,C). GUS staining of three *pGmHSFA2:GUS*-transformed *Arabidopsis* lines confirmed that *GmHSFA2* was expressed only in early-stage anthers of inflorescence during flowering (Figure 6D). The expression patterns of *GmHSFA2* under the HT condition (40°C) were evaluated by qRT-PCR using RNA samples extracted from flower buds of soybean, and the NT condition (30°C) was used as a control. The expression level of *GmHSFA2* increased significantly with time and peaked at the seventh day and then decreased rapidly after recovery with NT for 1 day (Supplementary Figure 12). This implies that *GmHSFA2* was extremely sensitive to HT during flowering in soybean reproductive tissues.

**FIGURE 7:**
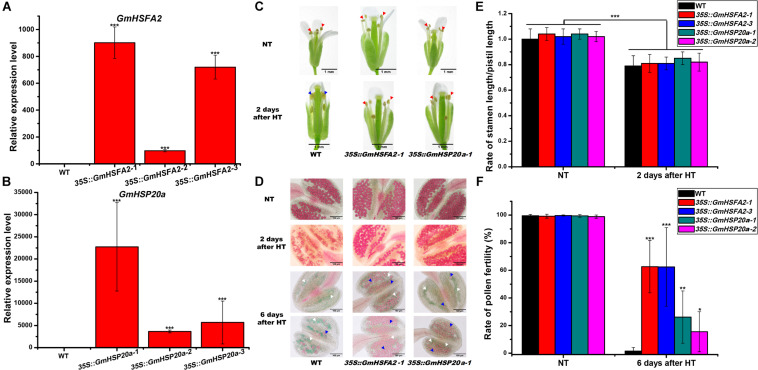
Effect of HT on male fertility in WT, *35S:GmHSFA2* and *35S:GmHSP20a* lines. **(A,B)** *GmHSFA2* and *GmHSP20a* expression levels in WT, *35S:GmHSFA2* and *35S:GmHSP20a* lines. The WT under NT condition was used as the control and qRT-PCR data are expressed as the mean values ± SD of three independent biological replicates. **(C)** Phenotype of anthers in WT, *35S:GmHSFA2* and *35S:GmHSP20a* lines under NT and HT conditions. The red and blue arrows indicate dehiscent and indehiscent anthers, respectively. **(D)** Phenotype of pollen in WT, *35S:GmHSFA2* and *35S:GmHSP20a* lines under NT and HT conditions. The red pollens pointed by blue arrows and green pollens pointed by white arrows indicate fertile pollens and sterile pollens, respectively. **(E,F)** Calculation of rate of stamen length/pistil length and rate of pollen fertility in WT, *35S:GmHSFA2* and *35S:GmHSP20a* lines under NT and HT conditions (*n* = 9). Asterisk indicates statistical differences, *P < 0.05; **P < 0.01; ***P < 0.001.

To further confirm the role of *GmHSFA2* in HT tolerance during flowering, three lines of *Arabidopsis* overexpressing *GmHSFA2* with different expression levels were selected for HT treatment (Figure 7A). In the HT tolerance assay (45°C for 3 days) during flowering, the top of the inflorescence of transgenic plants basically kept normal growth while that of the WT wilted (Supplementary Figure 13). Most importantly, HT treatment increased the stability of *35S:GmHSFA2* transgenic plants under HT stress (42°C for 4 h), which showed anther dehiscence and only a little pollen abortion after HT treatment for 2 and 6 days, respectively (Figures 7C,D), while the rate of stamen length/pistil length in both WT and transgenic lines decreased after 2 days of HT treatment (Figure 7E). However, the WT showed pollen shrinkage with anther indehiscence and male sterility (no pollen grains or most pollen abortion) after 2 and 6 days of HT treatment, respectively (Figures 7C,D,F).

**FIGURE 8:**
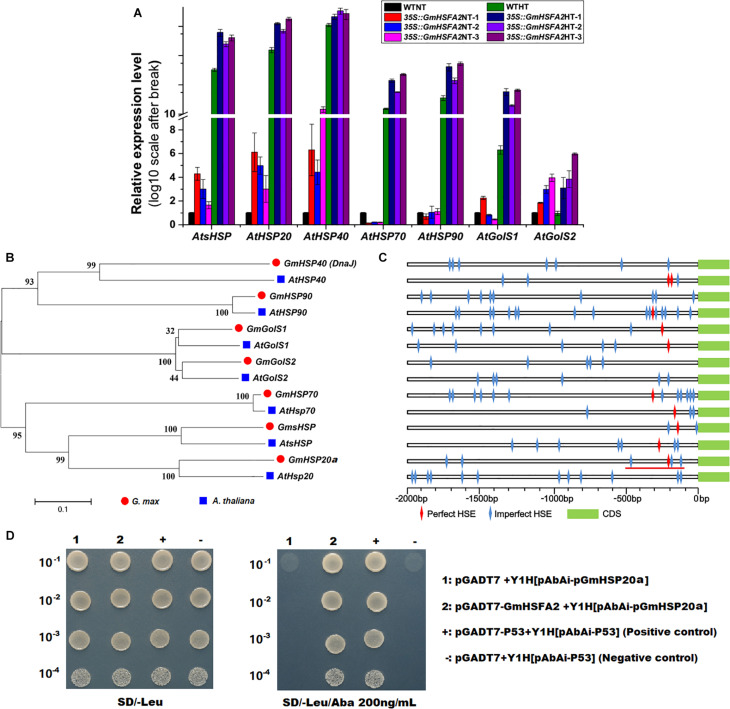
Effect of *GmHSFA2* on its downstream regulatory genes. **(A)** Expression levels of *HSP* and *GolS* in *35S:GmHSFA2 Arabidopsis* transgenic and WT plants under NT and HT conditions. The WT under NT condition was used as the control and qRT-PCR data are expressed as the mean values ± SD of three independent biological replicates. **(B,C)** Phylogenetic tree and schematic of *HSP* and *GolS*. The phylogenetic tree was constructed using MEGA 5.02 based on the NJ method. Bootstrap values in percentage (1000 replicates) are labeled on the nodes. Red line labeled cis-acting HSE motifs (–500 to –101 bp relative to the translational start codon of *GmHSP20a*) were used for cloning and Y1H assay. **(D)** The interaction between *GmHSFA2* and the promoter of *GmHSP20a* in yeast. The relevant bacterial solution was diluted in gradient of 1:10, 1:100, 1:1000, and 1:10,000, and grown on medium.

The expression levels of *GmHSFA2* downstream regulatory genes (*AtsHSP*, *AtHSP20*, *AtHSP40*, *AtHSP70*, *AtHSP90*, *AtGolS1*, and *AtGolS2*) under HT stress during flowering in *35S:GmHSFA2* plants were compared by qRT-PCR analysis. The transcripts of most of them except *AtHSP70* and *AtHSP90* were all higher than those in the WT under the NT condition (Figure 8A). After HT treatment, all of these downstream regulator genes were upregulated in *35S:GmHSFA2* plants compared with WT (Figure 8A). Moreover, the fold changes in the expression levels of almost all genes (except *AtHSP40*) between the two materials under HT were higher than that under the NT condition (Figure 8A). Most importantly, both RNA-seq data and qRT-PCR analysis showed that their homologous genes in soybean NF_1_ were upregulated by HT induction (Figure 8B and Supplementary Figures 11D–J).

Bioinformatics analysis showed that perfect and imperfect HSE motifs are distributed within promoter regions of selected *HSP*s and *GolS*s in both soybean and *Arabidopsis* (Figure 8C). Furthermore, we investigated a direct link between *GmHSFA2* and the promoter of a selected *GmHSP20a* (*Glyma.12G013100*) by Y1H assay (Figure 8D). In addition, the expression trend of *GmHSP20a* was consistent with that of *GmHSFA2* under HT stress (Supplementary Figure 12). Most importantly, the *35S:GmHSP20a Arabidopsis* transgenic lines also improved HT tolerance during flowering (Supplementary Figure 13 and Figures 7B–D,F). All these results show that *GmHSFA2* might improve the HT tolerance of soybean CMS-based F_1_ and transgenic *Arabidopsis* by regulating the expression changes of *HSP* and *GolS*.

## Discussion

The CMS-based hybridization method has been widely used in plant hybrid breeding due to its effective way of hybrid seed production by use of the CMS line, maintainer line, and restorer line. However, increasing evidence has indicated that male fertility of CMS-based F_1_ is affected by climate conditions such as HT stress (Zhao et al., 2009; Zhang et al., 2019; Nie et al., 2017). In this study, two soybean CMS-based F_1_ combinations, NF_1_ and YF_1_, were employed, and it was found that the male fertility of YF_1_ was obviously damaged by HT, such as anther indehiscence and decreased pollen fertility, thereby decreasing soybean yield (Supplementary Figure 1). Furthermore, RNA-seq and functional study of *GmHSFA2* were adopted to globally identify the DEGs and pathways participating in male fertility regulation of soybean CMS-based F_1_ under HT.

### Abnormal Anther/Pollen Development Is Related to Male Fertility Instability of HT-Sensitive F_1_ Under HT

In our RNA-seq, many *PL*, *PME*, *PG*, and *Exo-PG* genes showed differential expression between NF_1_ and YF_1_ under the HT condition (Figures 4A–C). Among them, pectinase (*PL*, *PME*, and *PG*) is a key enzyme involved in the degradation of plant pectin and participates in the regulation of anther/pollen development (Micheli, 2001; Ogawa et al., 2009; Corral-Martínez et al., 2016; Li et al., 2019). It has been shown that pectinase activity was decreased in anthers of Qx-115 (anther indehiscent phenotype material of chrysanthemum) during anther development (Li et al., 2019). Pectinase has been extensively studied in many plants. Wei et al. (2019) found that *PL*, *Exo-PG*, and *PME* were related to the fertility restorer of the CMS line in pepper. In *Brassica campestris*, downregulation of *BcPLL9* and *BcPLL10* results in disorder of pectin metabolism in pollen and finally leads to male semi-sterility (Jiang et al., 2014a, b). Also, in *B. campestris*, Huang et al. (2009) found that a *PG* gene (*BcMF2*) was specifically expressed in the tapetum and pollen and that its inhibition led to pollen deformity with abnormal intine development. Except *PL*s and *PG*s, *PME*s are also important for pollen development in plants. Recently, a CRISPR/Cas9 system-induced *BcPME37c* mutant has been characterized, and its mutation caused the abnormal thickening of the pollen intine in *B. campestris* (Xiong et al., 2019). The downregulation of pectinase genes in YF_1_HT may reduce the degradation of pectin, thus changing the maintenance of the anther wall, leading to anther indehiscence in YF_1_HT, and needs further research.

### Carbohydrate Undersupply and Sugar Transport Blockage Are Two of the Main Causes for Male Fertility Instability in YF_1_HT

**FIGURE 9:**
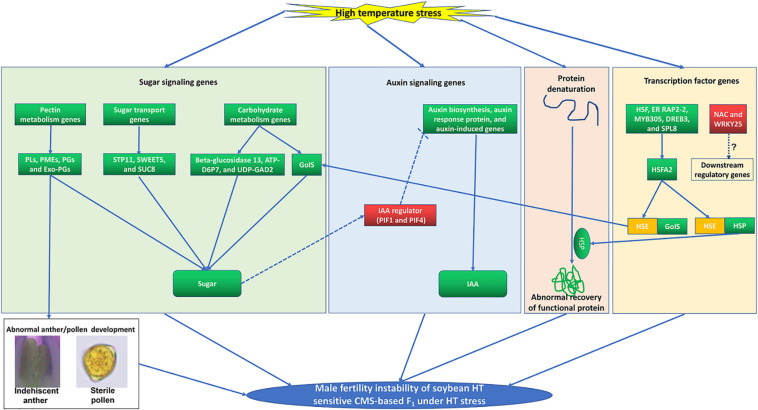
A proposed model incorporating the main processes that involved in the male fertility instability of soybean HT sensitive CMS-based F_1_ under HT stress. The up-regulated and down-regulated of gene or metabolite content are in red and green backgrounds, respectively.

Our RNA-seq analysis found that compared with NF_1_HT, the expression of hundreds of DEGs related to carbohydrate metabolism in YF_1_HT was downregulated, including *PL*s, *PME*s, and *beta-glucosidase* (Supplementary Table 7). In addition, many sugar transport-related DEGs, such as *STP11*, *SWEET5*, and *SUC8*, are also decreased in expression (Supplementary Table 7). Based on the determination of Suc, Glc, and starch contents, it is speculated that their reduction affected the male fertility of YF_1_ under HT stress (Figure 9). Moreover, similar results were found in tomato and cotton, where a decrease in sugar affected their male reproductive development under HT (Firon et al., 2006; Min et al., 2014).

Many studies have shown that genes related to either carbohydrate metabolism or sugar transport are associated with male sterility in plants. For example, our previous study found that male sterility of the soybean CMS line is associated with alterations in carbohydrate metabolism (Li et al., 2015). In cucumber, the downregulation of sugar transporters *CsHT1* and *CsSUT1* inhibits pollen germination and causes male sterility, respectively (Cheng et al., 2015; Sun et al., 2019). At the same time, they also protect against HT stress during pollen development (Frank et al., 2009; Min et al., 2013, 2014). Min et al. (2014) found that HT disrupted anther carbohydrate metabolism in cotton, including starch and Suc metabolism pathways, leading to abnormal male fertility development in H05 (HT-sensitive line) under HT. Further investigation demonstrated that *GhCKI* caused pollen abortion and anther indehiscence in cotton via inactivating starch synthase (Min et al., 2013). In tomato, HT-induced expressions of carbohydrate metabolism and sugar transport genes, such as sucrose phosphate synthase and sorbitol transporter, were involved in the HT response during pollen development (Frank et al., 2009). Thus, we speculate that carbohydrate undersupply and sugar transport blockage are two of the main causes for male fertility instability in YF_1_ under HT and need to be verified in future studies.

### Sugar Signaling-*PIF*-Auxin Signaling Pathway May Underlie Instability of Male Fertility in YF_1_ Under HT

Sugar plays a vital role as a protector defending against HT stress during male reproductive organ development (Min et al., 2013, 2014). And auxin is also closely related to instability of male fertility in plants under HT stress (Sakata et al., 2010; Higashitani, 2013; Min et al., 2014; Ding et al., 2017). In barley and *Arabidopsis*, HT can induce downregulation of genes related to auxin biosynthesis (*YUC2*, *YUC6*, and *TAA1/TIR2*), resulting in a sharp decrease in endogenous auxin level and eventually anther abortion (Sakata et al., 2010). Furthermore, exogenous auxin could completely reverse the male sterility of barley and *Arabidopsis* under HT stress (Sakata et al., 2010). Previous research has uncovered a pathway where sugar signaling is involved in plant growth by regulating auxin metabolism through the *PIF* protein (Stewart et al., 2011; Min et al., 2014). The *PIF* protein is an IAA regulator (Sun et al., 2012, 2019), and it is also involved in the HT response (Leivar and Quail, 2011). Furthermore, *PIF* expression was induced by low content of sugar, which altered auxin metabolism afterward and led to male sterility in cotton and cucumber eventually (Min et al., 2014; Sun et al., 2019). Similar to the cotton male sterility induced by HT stress, the sugar content and the expression levels of *PIF*s (*GmPIF1* and *GmPIF4*) in flower buds of YF_1_ were also altered under HT (Supplementary Table 8). Meanwhile, downregulated auxin signaling genes and content were observed in flower buds of YF_1_ under HT, indicating that *PIF* might act as a negative regulator of IAA biosynthesis, which is consistent with the results in *Arabidopsis* and cucumber (Sairanen et al., 2012; Sun et al., 2019). However, Min et al. (2014) showed that *PIF* acts as a positive regulator of HT-induced IAA biosynthesis in cotton. It appears that the sugar signaling-*PIF*-auxin signaling pathway acts as a master switch role during the male organ development under HT stress in soybean CMS-based F_1_, which needs further study (Figure 9).

### TFs Is Required for Enhanced Activation of HT Stress Response and Increased Thermotolerance in Soybean CMS-Based F_1_

Transcription factors are central regulators of gene expression affecting plant HT responses (Li et al., 2018). Many TF families, including *ER*, *MYB*, *DREB*, *SPL*, and *HSF*, are involved in HT stress response and enhanced tolerance in both model and crop plants (Hong et al., 2009; El-Kereamy et al., 2012; Wan et al., 2014; Chao et al., 2017; Li et al., 2018). In our study, some TF family members were upregulated in NF_1_HT related to YF_1_HT, including *GmER RAR2-2*, *GmMYB305*, *GmDREB3*, *GmSPL8*, and *HSFA2*, which may confer tolerance to NF_1_ under HT stress (Figure 5C and Supplementary Table 9). However, some TF family members may play as negative regulators, such as *GmNAC* and *GmWRKY25*, which were upregulated in YF_1_HT compared with NF_1_HT (Figure 5C). In *Arabidopsis*, a NAC-like gene (*AtAIF*) was found to be an inhibitor that controls anther dehiscence (Shih et al., 2014). Similarly, the overexpression of *GhWRKY22*, *GmWRKY45*, and *AtWRKY27* in *Arabidopsis* displayed the male fertility defect with decreased pollen viability (Mukhtar et al., 2017; Wang et al., 2019; Li et al., 2020). Most importantly, Dang et al. (2018) found that the overexpression of *CaWRKY27* in *Arabidopsis* inhibited the scavenging of H_2_O_2_ and played a negative regulator role in HT stress.

Although great progress has been made in deciphering the response of TFs such as *HSF* to HT stress in *Arabidopsis*, maize, tomato, tall fescue, and other plants (Charng et al., 2007; Giorno et al., 2010; Fragkostefanakis et al., 2016; Wang et al., 2017; Gu et al., 2019), few *HSF* genes have been elucidated in soybean, especially on the stability of male fertility. Four *GmHSFA2* genes (*Glyma.13G105700*, *Glyma.14G096800*, *Glyma.17G053700*, and *Glyma.17G227600*) were found in this study (Supplementary Table 10), and one of them (*Glyma.17G227600*) was overexpressed in *Arabidopsis*, showing the characteristics of HT and drought resistance during seedling in previous studies (Li et al., 2014). In this study, only *GmHSF-30* (*Glyma.14G096800* and *GmHSFA2* in this study) was induced by HT in soybean CMS-based F_1_ flower buds during flowering at the mRNA level (Supplementary Figure 11A). Fragkostefanakis et al. (2016) found that *HSFA2* is an important coactivator of *HSFA1a* during HT to control pollen viability by regulated *HSP101* and *HSP17.7C-CI* in tomato. In rice, *HSF* and *HSP* genes including *HSFA2a* and *HSP17.9A* are highly induced in HT-tolerant material rather in HT-sensitive varieties during anthesis under HT stress (González-Schain et al., 2016). In tomato, HT induced expressions of *HSFA2*, *sHSP* genes, *HSP70*, and *HSP101* during pollen development. In this study, both *HSFA2* and *HSP* (*sHSP*, *HSP20*, *HSP40*, *HSP70*, and *HSP70*) were induced by HT stress during flower bud development. Most importantly, a functional study found that *HSFA2* was directly involved in HT stress response and that inhibition of *HSFA2* reduces the viability and germination rate of tomato pollen under HT (Giorno et al., 2010; Fragkostefanakis et al., 2016). It has been reported that HT stress causes male sterility by affecting anther dehiscence and pollen production at a specific stage in *Arabidopsis* (Kim et al., 2001), and similar results were obtained in this study. Most importantly, ectopically expressing *GmHSFA2* enhanced HT tolerance in *Arabidopsis*, suggesting that it positively regulated HT tolerance during flowering in plants.

Our results suggest that *GmHSFA2* is a key regulator in response to HT stress. However, its regulatory molecular mechanism in soybean is still unknown. Many studies have shown that *HSF* promotes HT tolerance by binding to the HSE motifs in the promoter of *HSP* and *GolS* (Busch et al., 2005; Kotak et al., 2007; von Koskull-Doering et al., 2007; Fragkostefanakis et al., 2016; Wang et al., 2017; Gu et al., 2019). Frank et al. (2009) found that HT induced expressions of *HSF* and *GolS* during pollen development in tomato. However, the relationship among them during pollen development under HT stress is still unknown, especially in soybean. In our study, multiple *HSP* and *GolS* genes, including *sHSP*, *HSP20*, *HSP40*, *HSP70*, *HSP90*, *GolS1*, and *GolS2*, were upregulated by overexpression of *GmHSFA2* in *Arabidopsis* compared with WT under HT (Figure 8A). Most importantly, their homologous genes in soybean were also upregulated in NF_1_HT related to YF_1_HT, according to the RNA-seq and qRT-PCR analyses (Figure 5D). Furthermore, multiple HSE motifs were found in their promoters, and the Y1H assay revealed that there was a direct link between *GmHSFA2* and the promoter of *GmHSP20a*, indicating that *GmHSFA2* could regulate these genes (Figure 9). And *HSP* and helper molecular chaperones can help inactivated proteins reassemble into active high-level structures and maintain normal cell functions (Scharf et al., 2012). Most importantly, overexpression of *GmHSP20a* in *Arabidopsis* also conferred plant HT tolerance during flowering (Figures 7C,D,F). However, its HT tolerance was lower than that of *35S:GmHSFA2* transgenic plants under HT stress, indicating that *GmHSP20a* was only one of the downstream regulator genes of *GmHSFA2*. All the above results revealed that a complex TF regulatory network exists in soybean CMS-based F_1_ (Figure 9). As a key regulator in response to HT stress, the regulation mechanism of *GmHSFA2* in soybean needs to be explicated further.

## Data Availability Statement

The datasets generated by this study can be found in the NCBI using accession number PRJNA677945.

## Author Contributions

XD and SY conceived and designed the experiments. XD, QG, and QL performed the experiments. XD wrote the manuscript. SY and JG revised the manuscript. All authors read and approved the final manuscript.

## Conflict of Interest

The authors declare that the research was conducted in the absence of any commercial or financial relationships that could be construed as a potential conflict of interest.

## Footnotes

1 http://www.r-project.org/

2 http://www.geneontology.org/

3 http://www.kegg.jp/kegg/

4 https://www.soybase.org/

## Abbreviations

ATP-D6P7: ATP-dependent 6-phosphofructokinase 7
CMS: cytoplasmic male sterility
DEGs: differentially expressed genes
DREB: dehydration-responsive element binding
Exo-PG: exopolygalacturonase
Glc: glucose
GolS: galactinol synthase
ER: ethylene responsive
FPKM: fragments per kilobase of transcript per million mapped reads
GFP: green fluorescent protein
GO: Gene Ontology
GUS: β-glucuronidase
HT: high-temperature
HSF: heat shock factor
HSP: heat shock protein
KEGG: Kyoto Encyclopedia of Genes and Genomes
NJ: Neighbor joining
NT: Normal temperature
PCA: principal component analysis
PG: polygalacturonase
PIF: PHYTOCHROME-INTERACTING FACTOR
PL: pectate lyase
PME: pectin methylesterase
qRT-PCR: quantitative real-time PCR
RCA: repeated correlation analysis
SD: standard deviation
SPL: squamosa promoter-binding protein-like
Suc: sucrose
TFs: transcription factors
UDP-GAD: UDP-glucuronic acid decarboxylase
Y1H: yeast one-hybrid
WT: wild-type.
