# Supplementary material for: Comparative Transcriptomics Analysis and Functional Study Reveal Important Role of High-Temperature Stress Response Gene GmHSFA2 During Flower Bud Development of CMS-Based F1 in Soybean
Source: Front Plant Sci. 2020 Dec 15;11:600217. doi: 10.3389/fpls.2020.600217 (PMC7770188; doi:10.3389/fpls.2020.600217)
Supplement: Supplementary file 1 [file Data_Sheet_1.PDF]

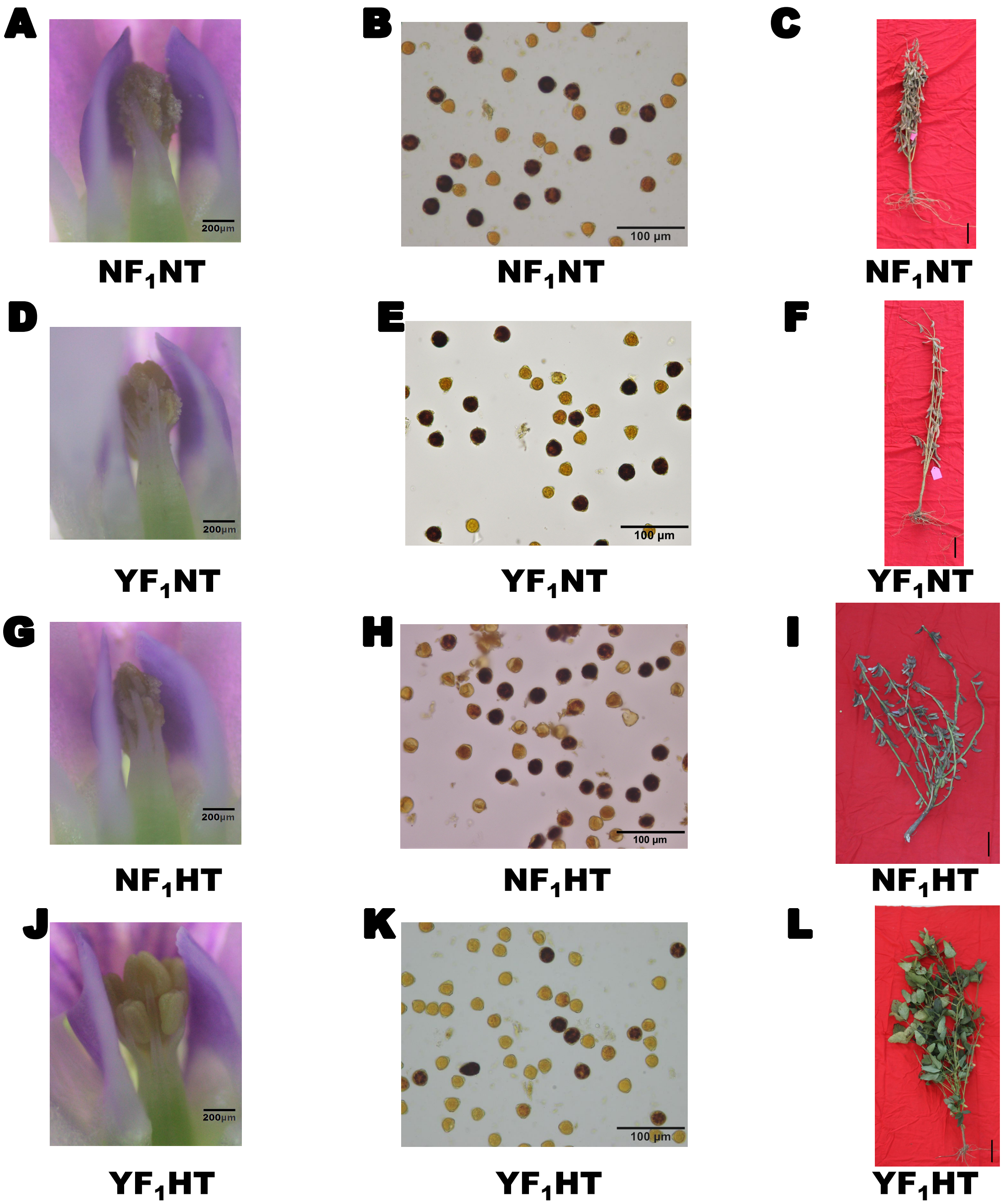

Figure S1 Phenotypic characteristics of soybean male fertility under NT and HT conditions in the field. **(A)** and **(D)**, Anthers from NF_1 and YF_1 under NT condition in the field. **(B)** and **(E)**, Pollens from NF_1 and YF_1 under NT condition in the field. **(C)** and **(F)**, Phenotypes of NF_1 and YF_1 at maturity under NT condition in the field. Scale bar: 6cm. **(G)** and **(J)**, Anthers from NF_1 and YF_1 under HT condition in the field. **(H)** and **(K)**, Pollens from NF_1 and YF_1 under HT condition in the field. **(I)** and **(L)**, Phenotypes of NF_1 and YF_1 at maturity under HT condition in the field. And YF_1 plants under HT showed as typical traits of semi-sterility, green leaves and some small, undeveloped young pods. Scale bar: 6cm. NT, 26~32℃/20~25℃(day/night) during flowering; HT, 32~38℃/24~28℃(day/night) during flowering.

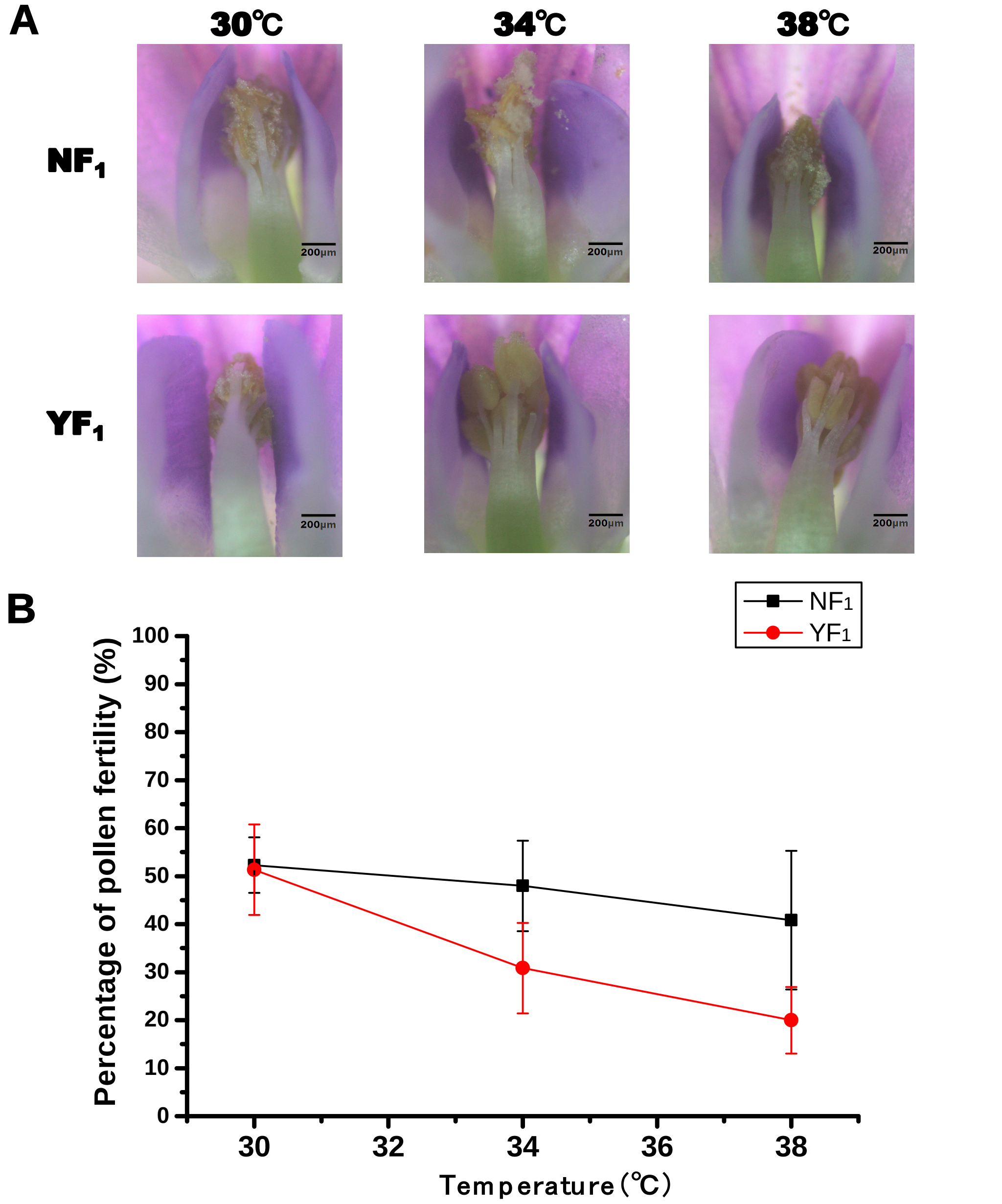

Figure S2 Soybean anther dehiscence and pollen fertility of NF and YF1 under gradient HT stress. **(A)** Anther dehiscence of NF and YF1 under gradient HT stress. **(B)** Pollen fertility of NF and YF under gradient HT stress.

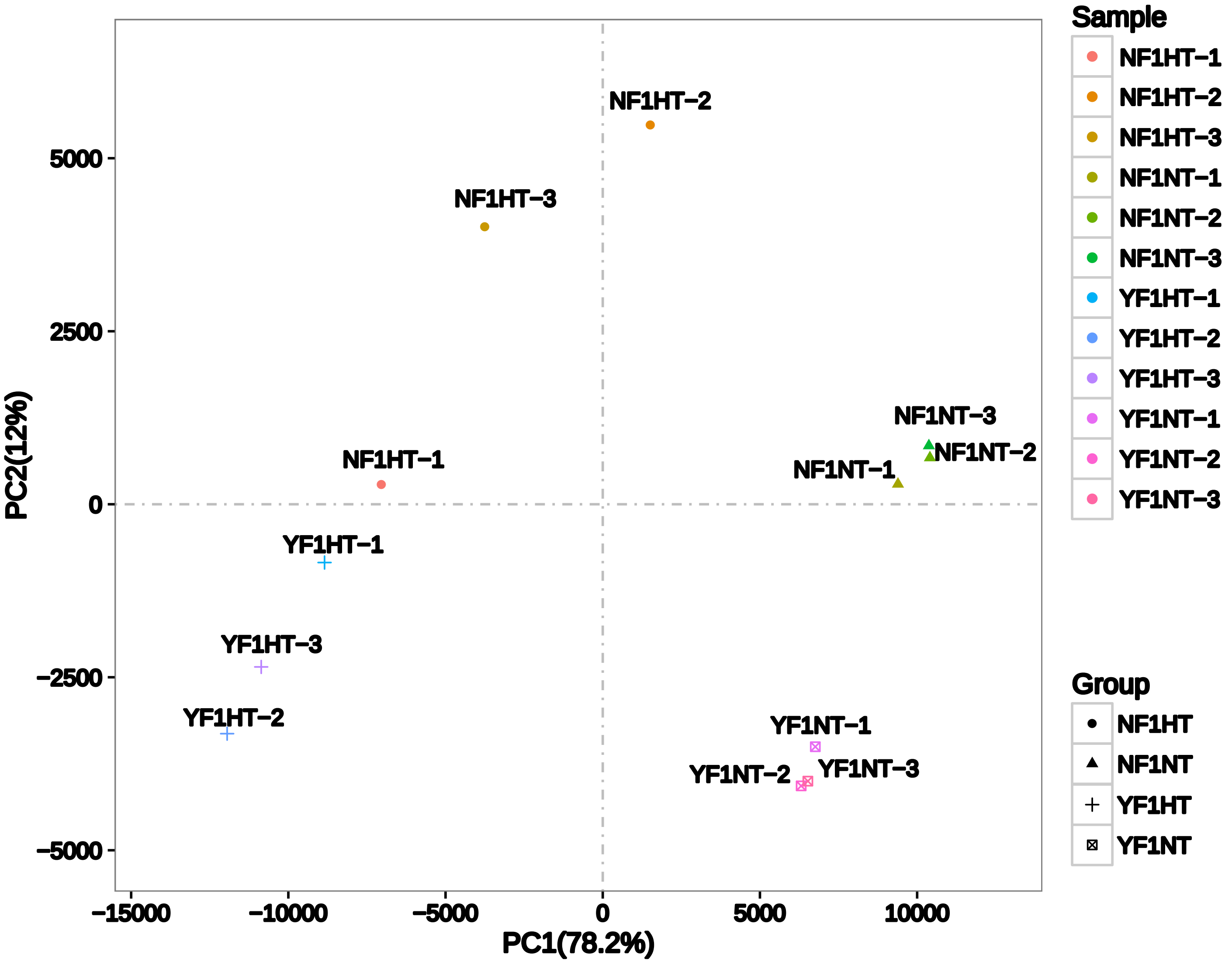

Figure S3 Principal component analysis score scatter plot for mass spectrum data of NF_1NT,YF_1NT,$NF_1HT$and YF_1HT. The x axis and y axis indicate the PC1 and PC2, respectively.

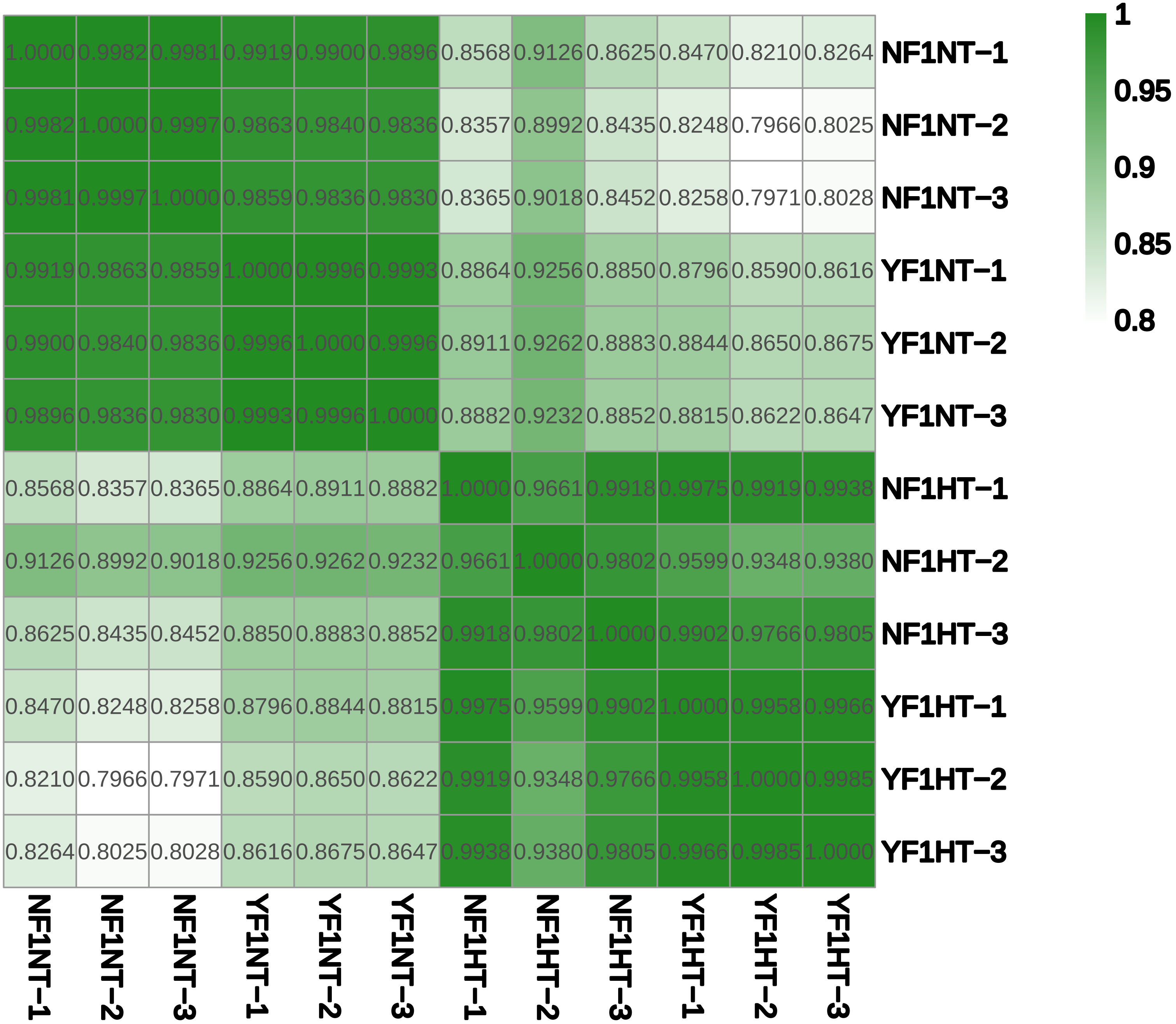

Figure S4 The repeated correlation assessment analysis of NF1NT, YF1NT, NF1HT and YF1HT.

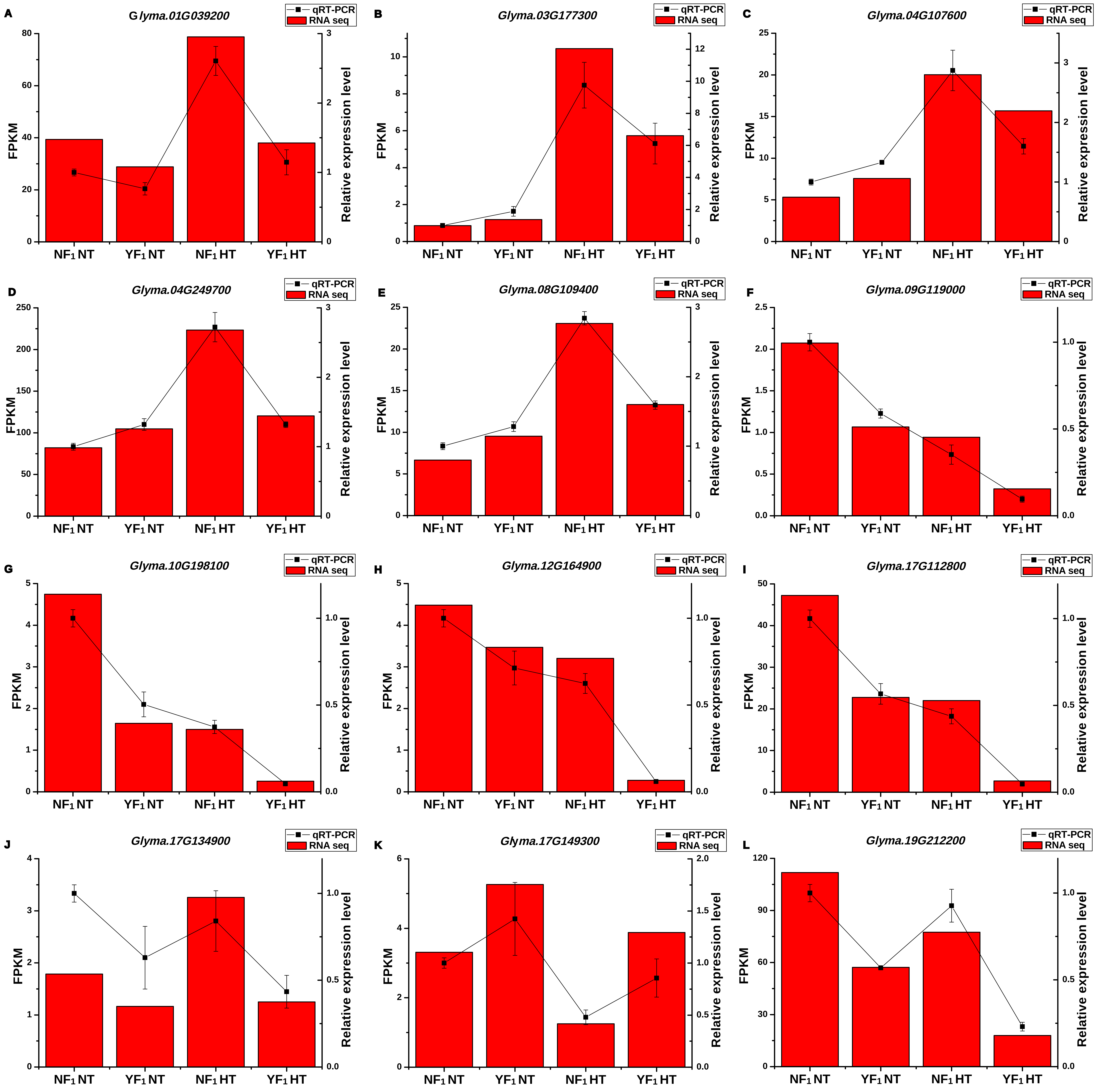

Figure S5 Expression levels of selected DEGs in NF_1NT, YF_1NT, NF_1HT and YF_1HT. The y-axis indicated the mRNA relative expression level generated from high-throughput sequencing and qRT-PCR analysis.The results were obtained from three biological replicates.

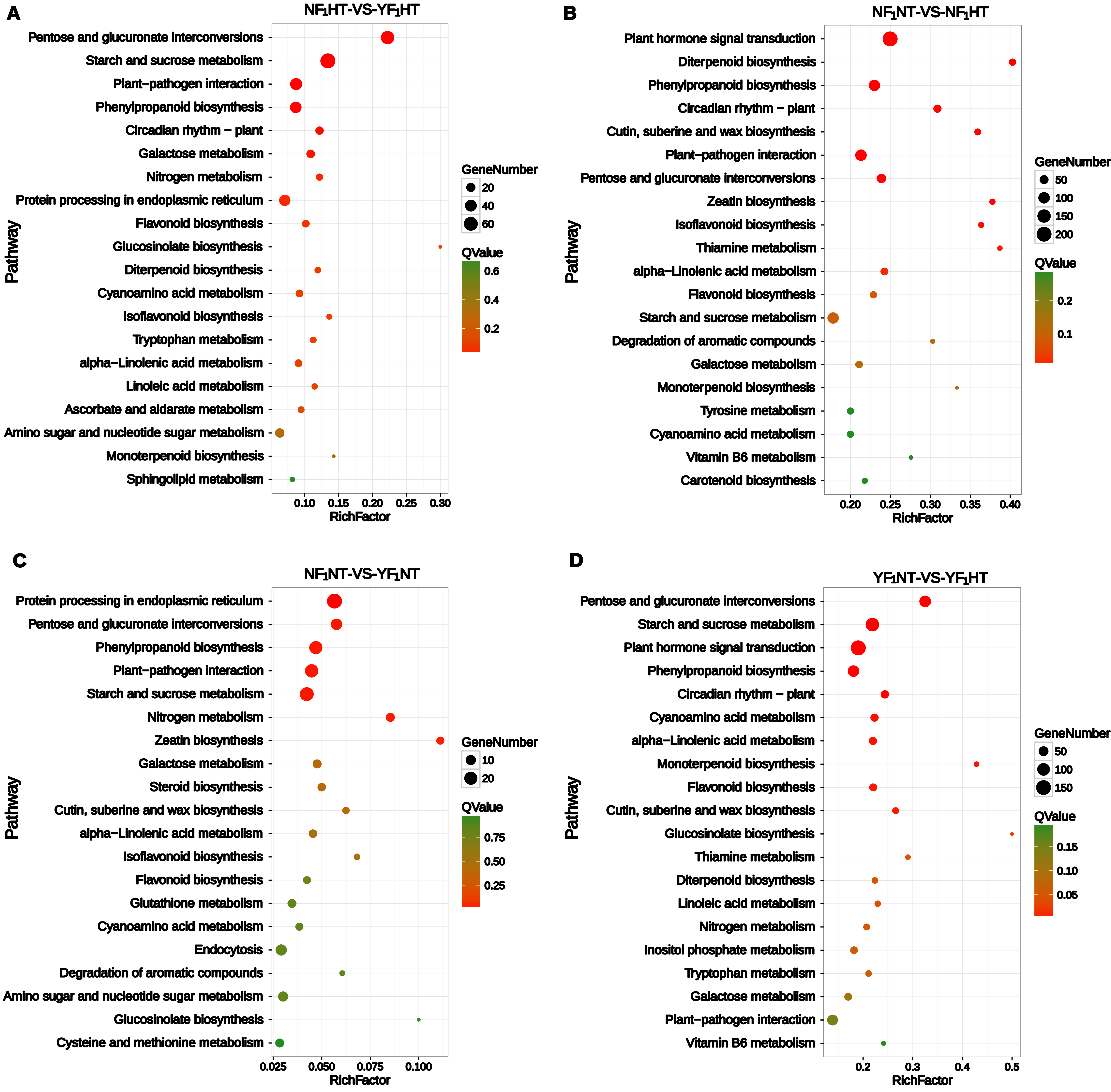

Figure S6 Top 20 of pathway enrichment for the the comparisons NF_1 HT VS NF_1 HT, NF_1 NT VS NF_1 HT, NF_1 NT VS YF_1 NT and YF_1 NT VS YF_1 HT, respectively. The x axis indicates the rich factor corresponding to each pathway, and the y axis indicates name of the KEGG pathway. The color of the point represents the p-values of the enrichment analysis. The size and color of bubbles represent the number and degree of enrichment of DEGs, respectively.

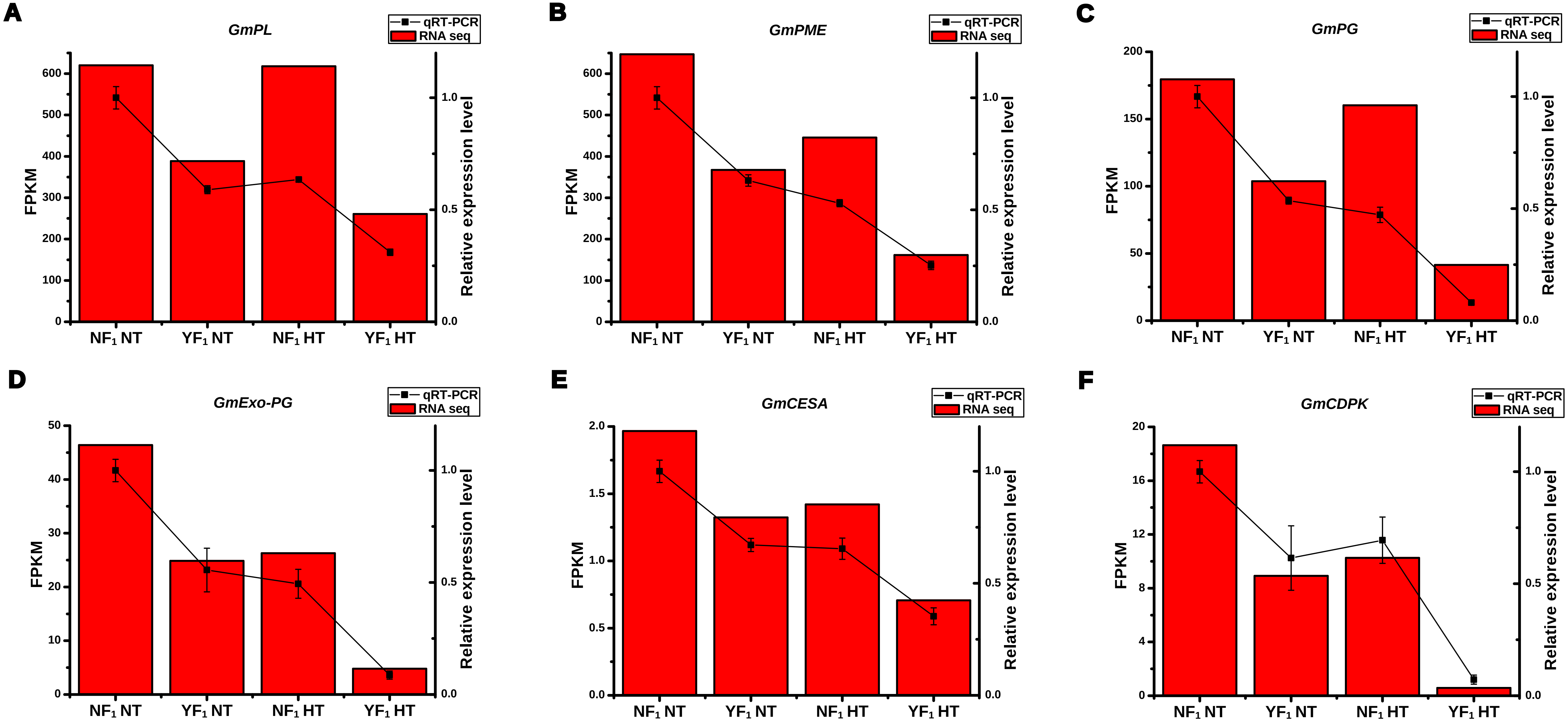

Figure S7 Expression levels of selected DEGs involved in anther/pollen wall development. The y-axis indicated the mRNA relative expression level generated from high-throughput sequencing and qRT-PCR analysis.The results were obtained from three biological replicates.

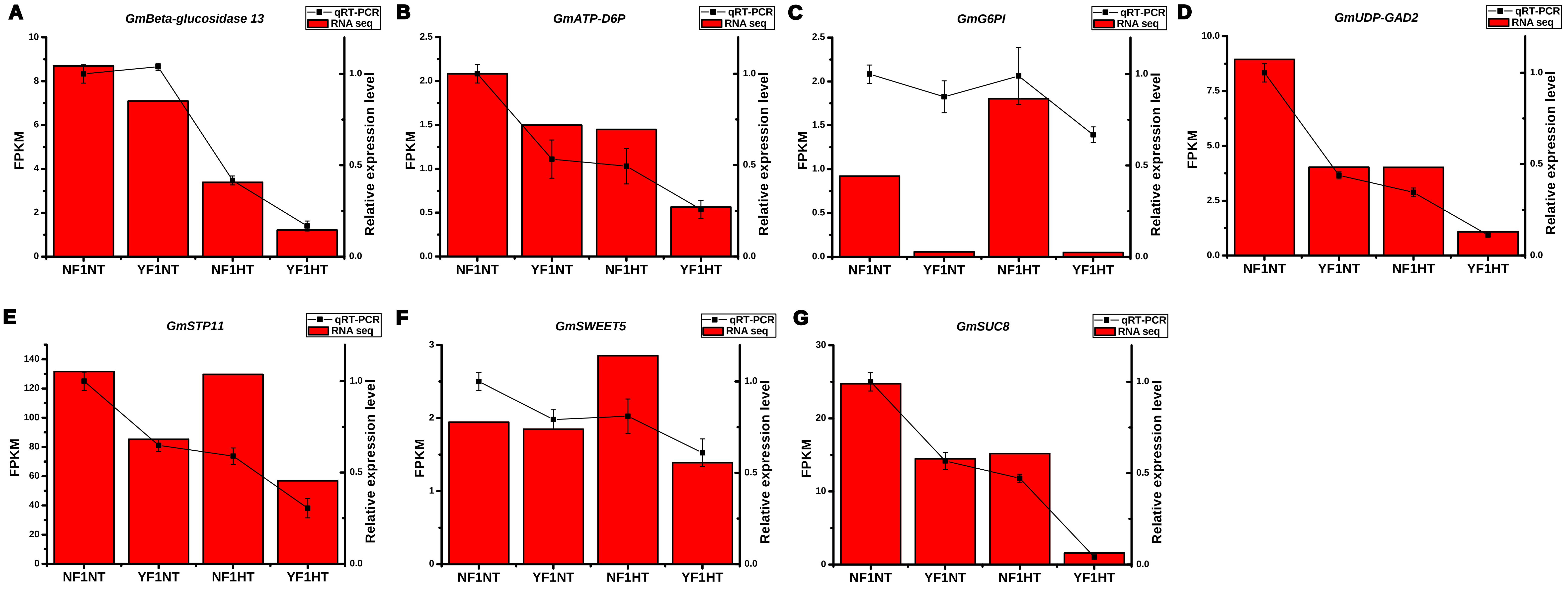

Figure S8 Expression levels of selected DEGs involved in carbohydrate metabolism and sugar transport. The y-axis indicated the mRNA relative expression level generated from high-throughput sequencing and qRT-PCR analysis.The results were obtained from three biological replicates.

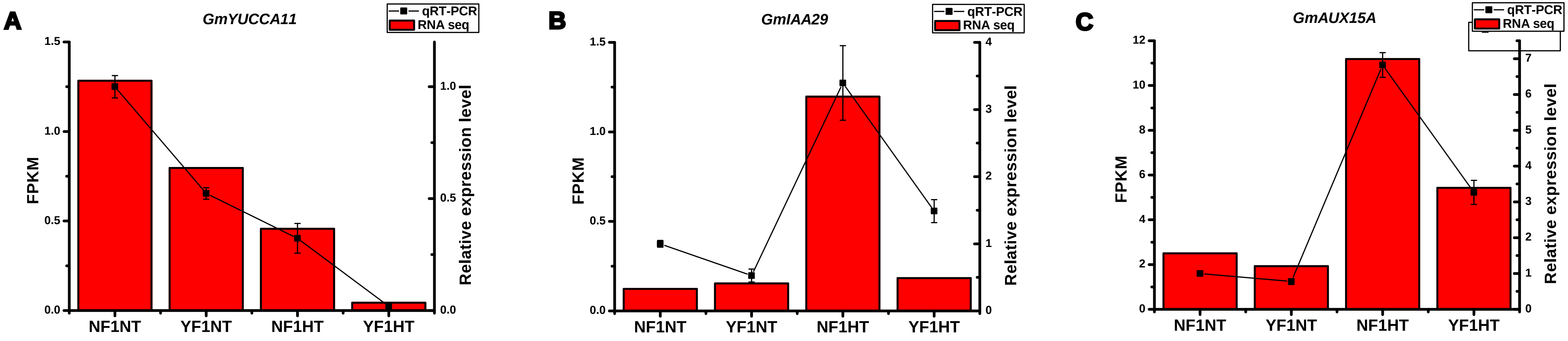

Figure S9 Expression levels of selected DEGs involved in auxin signaling. The y-axis indicated the mRNA relative expression level generated from high-throughput sequencing and qRT-PCR analysis.The results were obtained from three biological replicates.

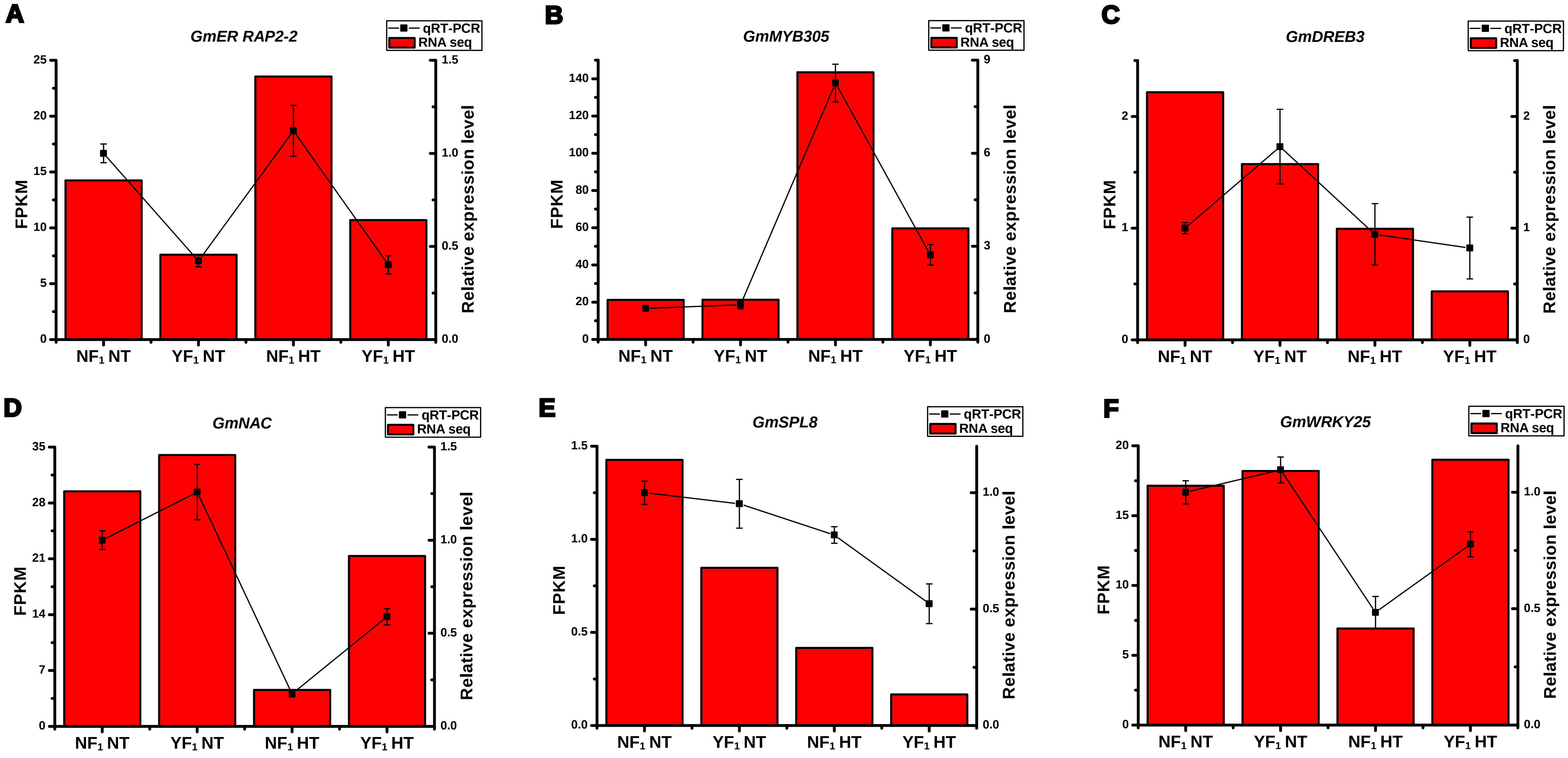

Figure S10 Expression levels of selected DEGs related to transcription factor. The y-axis indicated the mRNA relative expression level generated from high-throughput sequencing and qRT-PCR analysis.The results were obtained from three biological replicates.

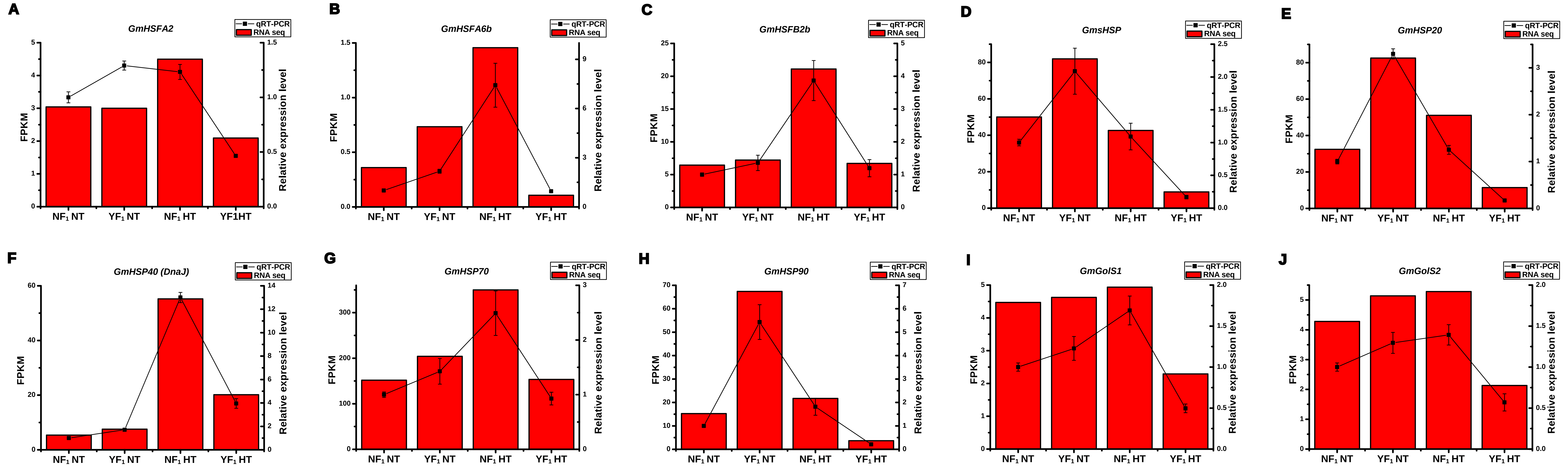

Figure S11 Expression levels of and its hypothetical downstream regulatory genes in soybean. The y-axis indicated the mRNA relative expression level generated from high-throughput sequencing and qRT-PCR analysis. The results were obtained from three biological replicates.

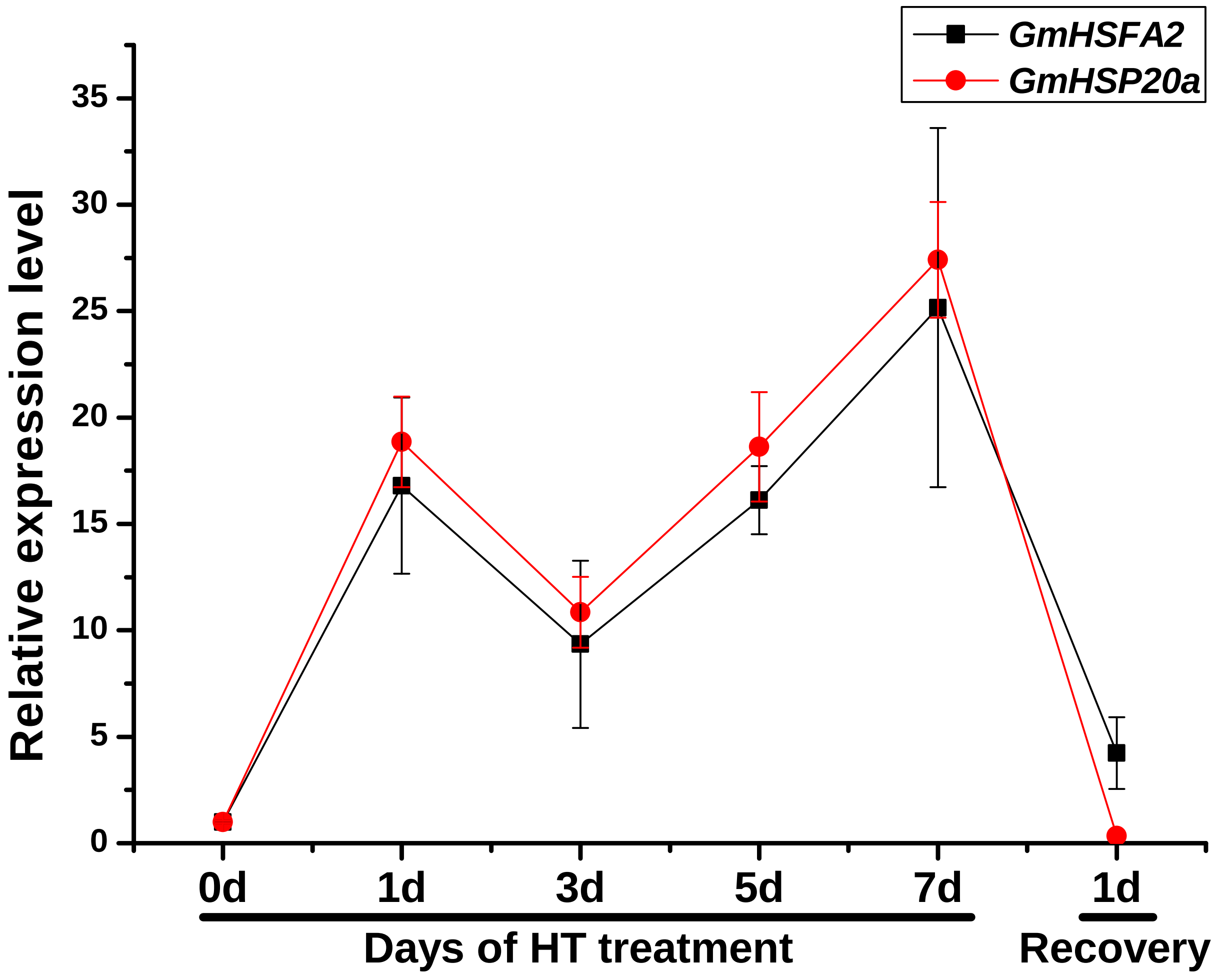

Figure S12 The expression patterns of *GmHSFA2* and *GmHSP20a* in mixed flower buds of soybean before and after HT treatment. The x-axis was the days of treatment, and the y-axis was the relative expression of *GmHSFA2* and *GmHSP20a*.

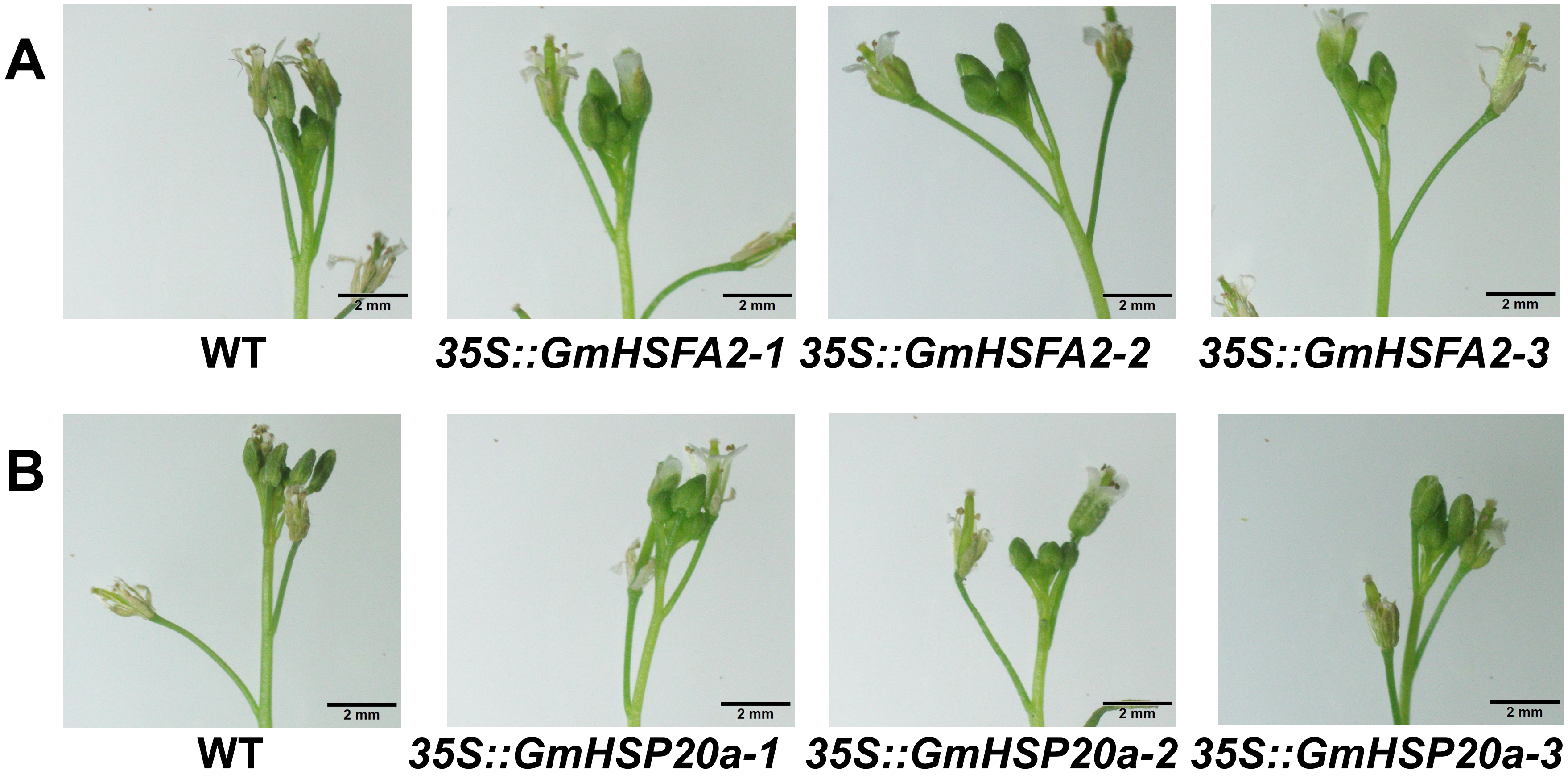

Figure S13 Over expression of *GmHSFA2* and *GmHSP20a* in *Arabidopsis* enhances plant HT tolerance during flowering. **(A)**, View of WT and *35S::GmHSFA2* plants grown under HT (45℃, 3 days). **(B)**, View of WT and *35S::GmHSP20a* plants grown under HT (45℃, 3 days).
